# Cryptococcal infection: host immunity, immune evasion and emerging immunotherapeutic strategies

**DOI:** 10.3389/fcimb.2025.1671873

**Published:** 2025-09-11

**Authors:** Fei Li, Xinxin Yu, Miao Li, Xiaoyu Ning, Kaijian Zhou

**Affiliations:** ^1^ Department of Pharmacy, The First Affiliated Hospital of China Medical University, Shenyang, China; ^2^ School of Pharmacy, China Medical University, Shenyang, China; ^3^ Center for Cell and Gene Therapy, The First Hospital of China Medical University, Shenyang, China; ^4^ Department of Plastic Surgery, The First Hospital of China Medical University, Shenyang, China

**Keywords:** *Cryptococcus*, cryptococcal infection, pattern recognition receptors, pathogen-associated molecular patterns, innate immune response, adaptive immune response, immune escape, immunotherapy

## Abstract

Cryptococcal infection is a typical opportunistic infection that significantly endangers human health, particularly to immunocompromised populations. As the top priority fungal pathogen listed by the World Health Organization, conventional antifungal drugs for cryptococcal infection are often ineffective and fail to completely eradicate the pathogen. One of the key factors underlying the treatment failure is the sophisticated immune escape strategies employed by *Cryptococcus*, which constitutes a major clinical challenge. Overcoming immune escape is key to improving therapeutic efficacy. Therefore, exploring new therapeutic methods, especially immunotherapy, is of paramount importance in combating the escape mechanisms and boosting the host’s defense capabilities. In this review, we focus on the host’s pattern recognition receptors, the innate and adaptive immune responses to the *Cryptococcus* infection, the immune escape tricks of *Cryptococcus*, and the prospects for immunotherapy, providing new insights for developing the anti-*Cryptococcus* immunotherapeutic strategies for the immunocompromised populations.

## 1 Introduction

*Cryptococcus* spp. is a kind of pathogenic fungus causing opportunistic infection, in which *Cryptococcus neoformans* and *Cryptococcus gattii* are the main pathogenic species. The *Cryptococcus neoformans* species complex includes *C. neoformans* and *C. deneoformans*, while the *Cryptococcus gattii* species complex comprises *C. gattii, C. bacillisporus, C. deuterogattii, C. teragattii*, and *C. decagattii* (Hagen et al., 2015). The distribution of *Cryptococcus neoformans* is widely and mainly within soil and bird guano, with 95% of the infected cases occurring in individuals with immunodeficiency, such as AIDS patients and organ transplant receptors. In outbreak areas (e.g. Vancouver Island), more than 90% of the cases with *C. gattii* infection occur in the immunocompetent people (Iyer et al., 2021; May et al., 2016). According to the most recent report, annual global deaths from cryptococcal meningitis reached approximately 147,000, with HIV-associated deaths accounting for 112,000 cases (Denning, 2024). *Cryptococcus neoformans* is the major etiology of fungal meningitis, posing a significant threat to the global public health, particularly to the immunocompromised populations (Casalini et al., 2024; Rajasingham et al., 2022). It is the first time that the World Health Organization has issued a checklist of the key pathogenic fungi in 2022, ranking *Cryptococcus* at the first position among the groups with the emergency priority (Casalini et al., 2024).

The polysaccharide capsule is the most distinctive structure feature of the pathogenic *Cryptococcus*, mainly composed of two kinds of polysaccharides, namely glucuronoxylomannan (GXM) and glucuronoxylomannogalactan (GXMGal) (Reese and Doering, 2003). Melanin is embedded in the matrix of the cell wall and forms an antioxidant barrier. Glucans, chitins, chitosans, mannoproteins (MPs), and Glycosylphosphatidylinositol (GPI)-anchored proteins, which are the important constitutes of the cell walls, are also pathogen-associated molecular patterns (PAMPs) (Garcia-Rubio et al., 2019; Gow and Lenardon, 2023). The PAMPs within cell walls are physically masked by the capsule. The damage of capsules and the exposure of PAMPs are required for the PAMPs to be recognized by host pattern recognition receptors (PRRs), which potentially lead to a harmful immune response, such as a Th2 bias. The interaction between the immune system of the host and *Cryptococcus* is highly intricate. On one hand, the innate and adaptive immunity of the host collaborate to recognize and eliminate *Cryptococcus*. Meanwhile, *Cryptococcus* employs various pathways to escape from the immune system of the host to survive intracellularly and to disseminate systemically, which ultimately leads to three clinical outcomes, including clearance or latency of *Cryptococcus*, chronic cryptococcal infection, and death of the infected subjects (Francis et al., 2024; Garcia-Rubio et al., 2019; Li et al., 2019).

Pharmacological therapy remains the sole method for clinical treatment of cryptococcal infection (Chang et al., 2024). Although this approach significantly reduced the acute-stage mortality, the drug toxicity in the kidneys and bone marrow was severe and the drug resistance was emerging constantly (Robbins et al., 2017). In addition, individuals with compromised immune function often fail to generate an effective immune response against *Cryptococcus*, although appropriate antimicrobial treatments were administered (Chang et al., 2024; Davis et al., 2019). Therefore, exploring new therapeutic strategies, especially those with protective effects on immunocompromised individuals, is of critical importance. This review focuses on the PRRs of host, the innate and adaptive immune responses against *Cryptococcus* invasion, and the prospects for immunotherapy, providing new insights for developing the immunotherapeutic approaches against *Cryptococcus* for immunocompromised individuals.

## 2 Pattern recognition receptors

The initiation for a host to establish an effective immune response against *Cryptococcus* is the binding of the PRRs on the innate immune cells to the PAMPs of *Crypto*coccus, serving as the first line of defense against infection (Campuzano and Wormley, 2018).

### 2.1 CLRs

**Figure 1:**
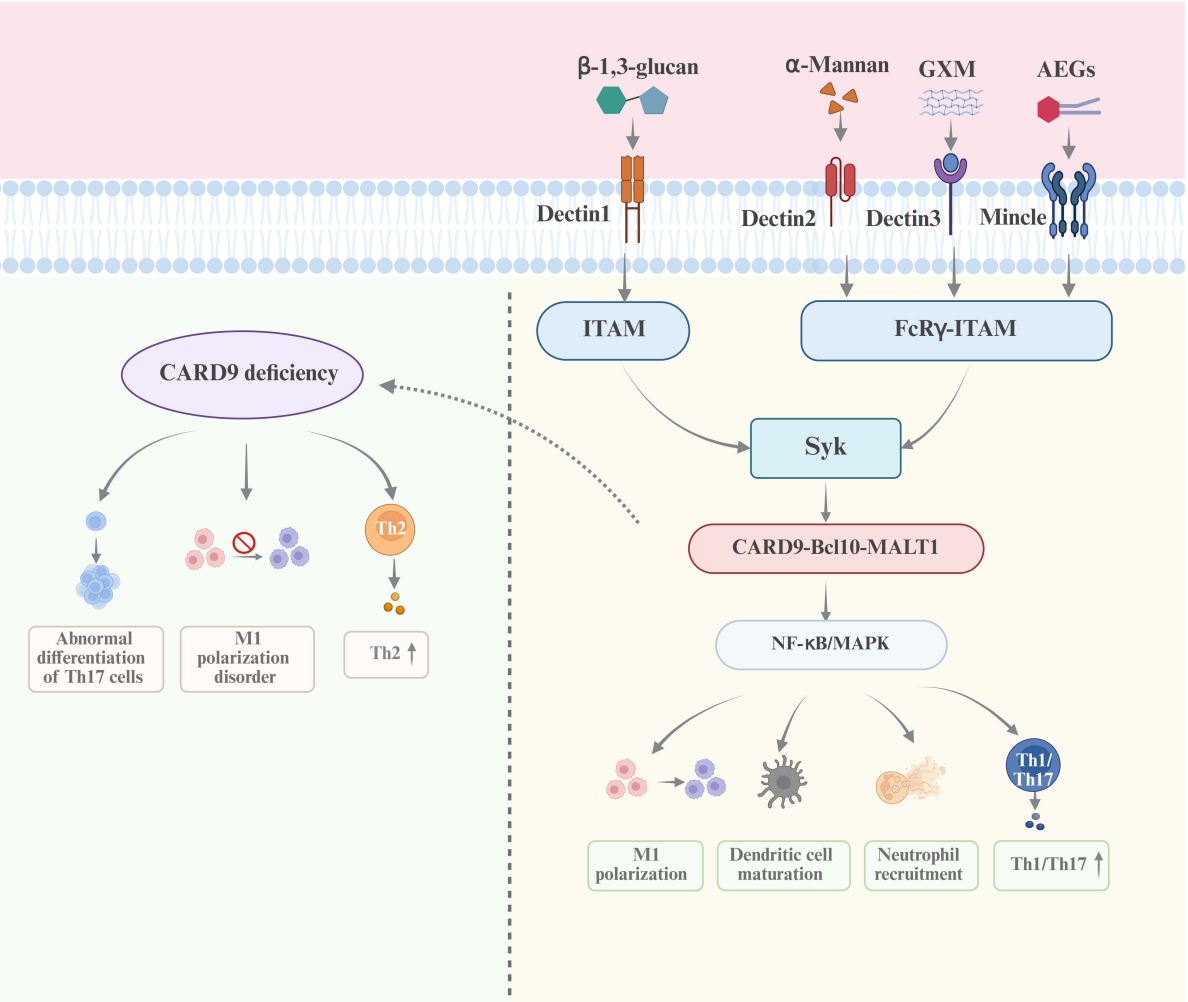
The recognition of CLRs to the PAMPs of *Cryptococcus* and the signaling pathways The CLRs, including Dectin1, Dectin2, Dectin3, MCL and Mincle, mainly recognize the polysaccharides of *Cryptococcus*, namely β-1,3-glucan, α-mannan and GXM. The Syk/CARD9/NF-κB/MAPK axis is the essential signaling pathway activated by CLRs-PAMPs interaction, in which CARD9 is the core molecule. The deficiency of CARD9 results in the incapability in the clearance of *Cryptococcus* infection.

The C-type lectin receptors (CLRs) primarily recognize the polysaccharides of *Cryptococcus* (Saijo and Iwakura, 2011; Speakman et al., 2020; Zhao et al., 2014). There are multiple studies directly implicating the crucial roles of CLRs in cryptococcal infection. Among them, Dectin-1 is able to recognize β-1,3-glucan. However, Dectin-1 is not indispensable for the host defense against *C. neoformans* infection due to the shielding effect of the polysaccharide capsule which covers the inner cell wall layer containing β-glucans(K. Nakamura et al., 2007). Studies have shown that the exposed β-1,3-glucan in *C. neoformans* can be recognized by Dectin-1. The phagocytic efficiency of Dectin-1^−/−^ macrophages is significantly lower than that of Dectin-1^+/+^ macrophages (Giles et al., 2009; Walsh et al., 2017). Upon binding with β-1,3-glucan, Dectin-1 recruits Syk kinase through the immunoreceptor tyrosine-based activation motifs (ITAMs). Syk phosphorylates the Syk-caspase recruitment domain-containing protein 9 (CARD9), promoting the formation of a CBM complex, consisting of CARD9, Bcl10 and MALT1. The CBM complex drives the nuclear entry of NF-κB and activates the MAPK pathway, thereby driving T cell differentiation and regulating the function of myeloid cells (Campuzano et al., 2020; Gross et al., 2006; Zhao et al., 2014). Additionally, Dectin-2 recognizes α-mannan and functions by activating the CARD9 signaling pathway through FcRγ-ITAM (McGreal et al., 2006). Studies on mice indicate that Dectin-2 may suppress the Th2 response and IL-4-dependent mucin production in the lungs after infection with *C. neoformans* (Y. Nakamura et al., 2015). Dectin-2^−/−^ dendritic cells are less effective in the phagocytosis of *C. neoformans* than Dectin-2^+/+^ dendritic cells (Kitai et al., 2021). The recognition domain of the Mincle receptor contains two hydrophobic pockets capable of binding long-chain fatty acids in addition to two sugar-binding pockets. This structural feature enables its efficient recognition of glycolipid pathogen ligands that interact with multiple binding sites. During *C. neoformans* infection, Mincle primarily recognizes acylated ergosterol β-glucoside (AEGs) through these hydrophobic pockets. Subsequently, Mincle initiates the CARD9 adaptor-mediated NF-κB pathway via ITAM signaling, inducing the secretion of proinflammatory cytokines and Th22-associated factor IL-22, thereby enhancing the host’s early immune response. However, its efficacy is limited as *C. neoformans* shields AEGs exposure through capsular polysaccharides, while other pattern recognition receptors (e.g., TLRs) can compensate for Mincle deficiency, attenuating its immunological effects (Sato et al., 2020; Watanabe et al., 2025). Dectin-3 can recognize directly the GXM of *C. neoformans* and *C. gattii*, and activate the NF-κB and ERK pathways to initiate the host defense response. Dectin−/− mice are highly sensitive to *C. neoforma*ns (serotype AD) and *C. gattii* (serotype B) infections, displaying increased fungal load in the lungs and weakened inflammatory response, which is not observed in other serotypes (Huang et al., 2018). Studies have demonstrated that CARD9 is a key molecule for inducing protective immunity against *Cryptococcus*. CARD9 deficiency leads to an abnormal differentiation of Th17 cells, a biased immune response towards Th2 type, impaired macrophage function, and the loss of the ability to clear *C. neoformans* infection (Campuzano et al., 2020). The signaling pathways activated by the recognition of CLRs to the cryptococcal PAMPs are shown in **Figure 1**. These findings provide new insights into the immune mechanism of cryptococcosis and offer potential targets for immunotherapy strategies against fungal infections.

### 2.2 TLRs

Toll-like receptors (TLRs) are type I transmembrane PRRs (Nakamura et al., 2008). TLR2 recognizes sterylglucosides and MP88 of both *C. neoformans* and *C. gattii* and promotes the secretion of TNF-α and IL-6 via the MyD88/NF-κB/MAPK pathway, enhancing the phagocytic activity of macrophages and the activation of γδ T cells (Normile et al., 2022; Rella et al., 2015). Notably, TLR2-deficient γδ T cells do not produce IL-17A, giving rise to a total loss of protective effectiveness of the sterylglucosidase 1 (sgl1) deletion (sgl1Δ) vaccine (Normile et al., 2022). Recent studies have shown that the absence of TLR4 signaling in *C. neoformans* enhances non-specific phagocytosis by upregulating macrophage scavenger receptor 1 on macrophages, a process involving the FcyRII/III and Syk signaling pathways (Onyishi et al., 2023). Additionally, both *C. neoformans* and *C. gattii* are able to activate bone marrow-derived dendritic cells through the TLR9-MyD88 signaling pathway and promote Th1 type immune response. The deficiency of TLR9 weakens the host’s ability to clear the two kinds of *Cryptococcus*, leading to higher fungal loads and poorer survival rates (da Silva-Junior et al., 2021; K. Nakamura et al., 2008; Y. Zhang et al., 2010). In *C. neoformans* infection, the absence of TLR9 mainly affects the recruitment of Th1 cells and IFN-γ production (Y. Zhang et al., 2010), while in *C. gattii* infection, the lack of TLR9 diminishes the Th1/Th17 immune response, leading to a significant increase in titan cells and the spread of the pathogen (da Silva-Junior et al., 2021; Yang et al., 2022). Therefore, TLR9 is one of the core receptors of the host and plays a crucial role in the elimination of the cryptococcal infection. Overall, the effect of TLR ligands as adjuvants may be more effective than those as direct vaccine targets.

### 2.3 NLRs

NOD-like receptors (NLRs) are positioned in cytoplasm. *C. neoformans* capsular mutant (such as *cap59*Δ) can activate the NOD-like receptor family pyrin domain containing 3 (NLRP3) pathway(C. Guo et al., 2014). The NLRP3 deletion weakens the IL-1β secretion and the neutrophil recruitment, indicating that regulating the NLRP3 inflammasome pathway may help defend the infection of *C. neoformans* in patients (Franchi et al., 2012; Lei et al., 2013). The alteration of the intracellular ion concentration (such as potassium ion efflux) and the production of reactive oxygen species (ROS) caused by cryptococcal infection can activate the NLRP3 inflammasome and enhance the immune response (Dostert et al., 2008; Pétrilli et al., 2007).

### 2.4 SRs

Scavenger Receptors (SRs) are featured by the capability of recognizing polysaccharides with negative charges (such as phosphorylated mannan) on the surface of *Cryptococcus*, mediating the non-macrophage-dependent (non-specific) phagocytosis and the clearance of *Cryptococcus* (Onyishi et al., 2023; Pathakumari et al., 2020). Members of the scavenger receptor family, Scavenger Receptor Class F Member 1, recognizes β-1,3-glucan and initiate endocytosis of *C. neoformans*, resulting in the formation of the phagosomes. CD36, a Class B Scavenger Receptor, binds to membrane phospholipids (such as phosphatidylinositol) and inhibits the intracellular replication of *C. neoformans* (Means et al., 2009). Mice with double knockout of Scavenger Receptor Class F Member 1 and CD36 exhibited 100% mortality after 7 days of *C. neoformans* infection (Pathakumari et al., 2020). Macrophage scavenger receptor 1 recognizes the glucuronic acid group of GXM and co-localizes with TLR4, synergistically activating the Vav/Rac1 pathway to mediate phagocytosis (Onyishi et al., 2023). Macrophages recognize the exposed β-1,3-glucan of *C. neoformans* through their collagen structure receptor, macrophage receptor with collagenous structure (MARCO). In hepatic macrophages, MARCO maintains the stability of actin by inhibiting ROCKII kinase, thereby blocking the “vomocytosis” that allows *C. neoformans* to escape from macrophages. The absence of MARCO leads to systemic dissemination of *C. neoformans* and a 10-fold increase in the level of *C. neoformans* within the brain (Onyishi et al., 2024; J. Xu et al., 2017). CD5 is expressed by lymphocytes and recognizes β-1,3-glucan, which can provide co-stimulatory signals and enhance the activation of T and B cells as well as the production of cytokines (such as IFN-γ, TNF-α, IL-6, and IL-12), promoting the differentiation of Th1/Th17 cells and the production of anti-*C. neoformans* antibodies IgG2c. CD5-deficient mice exhibit defects in Th1/Th17 differentiation, resulting in delayed *C. neoformans* clearance. Soluble human CD5 recognizes β-glucan through its SRCR domain, enhancing TNF-α/IFN-γ production by macrophages and thereby driving M1 polarization. Simultaneously, Soluble human CD5 inhibits the function of regulatory T cells, reversing the immunosuppressive microenvironment (Velasco-de-Andrés et al., 2021; Velasco-de Andrés et al., 2020). Additionally, it has been reported that Dectin-2 plays a pivotal role in the phagocytosis of *C. neoformans* by bone marrow-derived dendritic cells, potentially facilitating actin polymerization and phagocytic activity through the CARD9 and Syk-PI3K signaling pathways (Kitai et al., 2021).

### 2.5 NKG2D

The latest research has found that natural killer cell group 2D receptor (NKG2D) is a PRR that directly recognizes the polysaccharide ligands on the surface of *C. neoformans*, which activates the degranulation of natural killer (NK) cells and T cells to degranulate and the killing of fungi (Charpak-Amikam et al., 2024). Flow cytometry (FCM) detection confirmed that the NKG2D-IgG-Fc fusion protein can bind to the surfaces of various fungi, including *C. neoformans*, although the chemical structure of its fungal ligands has not yet been deciphered. In NKG2D-deficient mice infected by *C. neoformans*, the levels of *C. neoformans* in the lungs and brain increased threefold, and the survival rate decreased by 50%, confirming the protective role of NKG2D. The immunological intervention strategies targeting NKG2D, such as agonists or adoptive cell therapy, may become a novel direction for antifungal treatment (Charpak-Amikam et al., 2024).

## 3 Innate immune response

The innate immune response is the first line of defense against *Cryptococcus*, and its efficacy directly affects the progression and outcome of the infection (Heung, 2017). However, *Cryptococcus* has also evolved immune evasion strategies (Yang et al., 2022; Zaragoza, 2019). A detailed analysis of the interactions between the innate immune response and the cryptococcal infection not only reveals the complex mechanisms of host-*Cryptococcus* interaction, but also provides a theoretical foundation for the development of targeted immunotherapies.

### 3.1 Macrophages

Macrophages are crucial innate immune cells during the course of cryptococcal infection. Through multiple PRRs, such as Dectin-1, TLR2/4 and CD36, macrophages recognize β-1,3-glucan or GXM, initiating the engulfment of *Cryptococcus* (Campuzano and Wormley, 2018; Tucker and Casadevall, 2002). There are two polarization forms of macrophages, type M1 and M2 (Conn and Wozniak, 2023).

M1 macrophages are induced by IFN-γ and TNF-α, functioning as the major fighters against cryptococcal infection by the following mechanisms. Firstly, M1 macrophages induce inducible nitric oxide synthase (iNOS) to generate nitric oxide (NO) and ROS, which damage the ergosterol and chitin synthase within the cellular membrane of *C. neoformans*. Secondly, M1 polarization (iNOS^+^/arginase 1^-^ (Arg1^-^)) is stimulated through NO-activated signal transducer and activator of transcription 1 (STAT1) signaling. Thirdly, the blockage of the expression of *C. neoformans* effector protein, Cryptococcal Protein Linked to Virulence 1 (Cpl1), inhibits M2 polarization mediated by TLR4/STAT3 (Dang et al., 2022; Y. Wang et al., 2022). Lastly, TNF-α and IL-12 are released to induce the Th1 immune response and IFN-γ production. IFN-γ maintains the M1 phenotype (iNOS^+^/Arg1^-^) of macrophages through activating the STAT1 pathway (Y. Wang et al., 2022). STAT1 knockout mice show a significant increase in fungal load and Arg1 expression, and a loss of bactericidal capacity (Bryan et al., 2021; Leopold Wager et al., 2018; Marina et al., 2025). IFN-γ pre-stimulation can confer a “memory-like” phenotype to macrophages, enhancing the rapid killing response during secondary infection (Leopold Wager et al., 2018). Additionally, *C. gattii* exhibits strain-specific strategies to evade clearance: high-phagocytic strains induce M1 depletion and mitochondrial tubulation, enabling intracellular dormancy and persistence, while low-phagocytic strains trigger robust M1 responses (Voelz et al., 2014; Yang et al., 2024b). This distinct strategy enables the pathogenic *C. gattii* to establish a persistent infection within macrophages, which is not observed in *C. neoformans*.

M2 macrophages, induced by IL-4 and IL-13, are the “accomplices” of cryptococcal immune escape of *C. neoformans.* The mechanisms include (1) high level of the Arg1 expression, consuming arginine which is needed for the production of NO and inhibiting NO production (Bryan et al., 2021; Hansakon et al., 2023; Marina et al., 2025), (2) secretion of IL-10 and TGF-β, inhibiting the inflammatory response and providing an intracellular survival environment for *C. neoformans* (Bryan et al., 2021; Hansakon et al., 2023; Leopold Wager and Wormley, 2014; Marina et al., 2025), and (3) proliferation of *C. neoformans* intracellularly, spreading to the central nervous system by the “Trojan horse” pathway (Bryan et al., 2021; Hansakon et al., 2023; Leopold Wager and Wormley, 2014; Marina et al., 2025).

Recent studies have reported that immune metabolic reprogramming regulates the polarization of macrophages from M1 to M2. Macrophages mainly rely on glycolysis to maintain the M1 phenotype, while depend on fatty acid oxidation to maintain the M2 phenotype (Marina et al., 2025). Upregulation of macrophage Arg1 expression and fatty acid oxidation through GXM and Cryptococcal Protein Linked to Virulence 1 is an important pathway for *C. neoformans* escape from the phagocytosis and clearance by M1 macrophages. Notably, targeting mitochondrial vulnerabilities in *C. gattii* presents a promising approach to prevent persistent dormancy. These findings may establish novel research directions for developing anti-cryptococcal immunotherapy strategies.

### 3.2 Dendritic cells

In the cryptococcal infection, dendritic cells (DCs) are the most pivotal antigen presenting cells and exhibit the greatest potential for T cell activation (Ramirez-Ortiz and Means, 2012). The interaction between *Cryptococcus* and DCs is a dynamic competition between the host’s protective immunity and the cryptococcal immune escape. DCs recognize *Cryptococcus* through PRRs and initiate T cell immunity, while *Cryptococcus* inhibits the activities of DCs by capsules and other virulence factors to survive. DCs recognize the MPs of *Cryptococcus* through the MRs, mediating the endocytosis and lysosomal degradation of the pathogen. Furthermore, cooperating with the TLR9-MyD88 pathway activated by cryptococcal DNA, the interaction between MRs and MPs promotes the migration of DCs to the lymph nodes and the antigen presentation to T cells, ultimately activating Th1/Th17 cells and secreting protective factors such as TNF-α/IFN-γ. Meanwhile, the capsule GXM/GXMGal blocks the maturation of DCs, downregulates the expression of MHC-II and CD80/CD86 on DCs, which inhibits the proliferation of T cells (Conn and Wozniak, 2023; Goughenour et al., 2023; Wozniak, 2018). Additionally, after the endocytosis into DCs, the *Cryptococcus* enters lysosomes and is killed by enzymes such as ROS and cathepsin B, which have the ability to destroy the cell wall of *Cryptococcus* by the way of osmotic lysis (Conn and Wozniak, 2023; Wozniak, 2018). In particular, *C. gattii* can suppress DC maturation and the T cell pathways, overcome the physical barrier of a cage-like structure formed by the phagosomal filamentous actin as well as break the inhibition of the TNF-α signaling pathway. The unique evasion strategy of *C. gattii* may explain the high pathogenicity of *C. gattii* in immunocompetent hosts (Huston et al., 2013; Jamil et al., 2020).

TNF-α stabilizes the polarization of DC1 through epigenetic mechanisms and promotes protective Th1 and Th17 immune responses. Reduced TNF-α secretion enhances murine susceptibility to *C. neoformans* infection by triggering alternative activation of DCs (Eastman et al., 2019). The recent studies have found that basic leucine zipper transcription factor ATF-like 3 (Batf3)-dependent conventional dendritic cells 1 (cDC1) play a key role in anti-*C. neoformans* infection by promoting Th1 polarization. cDC1 deficiency significantly decrease the activation of CD4^+^ T cells in lung and brain and the secretion of Th1 cytokines (such as IFN-γ and TNF-α), resulting in the increase in fungal load (Coelho, 2024; J. Xu et al., 2024). The latest research reports that Batf3-dependent cDC1 significantly upregulates genes related to T cell recruitment and Th1 polarization during infection, such as IL-12b, Stat4, and Ccl22, and produces high levels of IL-12, thereby enhancing the immune response and promoting *C. neoformans* clearance (Coelho, 2024; J. Xu et al., 2024). In *C. gattii* infection, there are differences in the responses of different DC subpopulations. Although monocyte-derived DCs can effectively phagocytose and kill *C. gattii*, only the cDC1 subpopulation can produce sufficient IL-12 to drive Th1 polarization (Jamil et al., 2020). The strategy of promoting Th1 and Th17 cell immune responses through DCs provides new ideas for designing vaccines and immunotherapies against cryptococcal infection (Hole et al., 2019).

### 3.3 Neutrophils

Neutrophils play a key role in defending cryptococcal infection at the early stage. The neutrophils recruited to the lungs exhibit greater antimicrobial capacity than those of the macrophages (Diamond et al., 1972). Migration and aggregation to the site of infection are essential for neutrophils to eliminate *Cryptococcus* at the early phase of infection, and the following mechanisms are involved. First, the paracrine pathway of complements is activated, producing strong chemokines, such as C3 and C5a, which chemotactically orientate neutrophils to migrate to the infection sites (Sun et al., 2016). The strain of *C. gattii* R265 can be recognized by neutrophils through the complement C3-mediated opsonization. However, the intensity of the C5a-C5aR signal induced by *C. gattii* is significantly weaker than that of the strain of *C. neoformans* H99, resulting in insufficient upregulation of CD11b. This is one of the reasons that *C. gattii* can be pathogenic in immunocompetent hosts in clinical settings (Ueno et al., 2019). Second, the C5a/C5aR signalling upregulates the CD11b expression of neutrophils and promotes Mac-1 (CD11b/CD18) binding to Intercellular Adhesion Molecule-1 (ICAM-1) of vascular endothelial cells, which mediates trans-endothelial migration of neutrophils to the infection loci. Blockade of CD11b nearly completely inhibits the intravascular migration and cryptococcal killing ability of neutrophils (Filippi, 2019). Third, the production of neutrophil extracellular traps and ROS directly captures and kills *C. neoformans* and *C. gattii*. However, the efficiency of NETs in wrapping and clearing C. gattii is lower as compared to that of *C. neoformans*. Moreover, the strain of *C. gattii* R265 displays stronger ability in resisting oxidative stress than that of the strain of *C. neoformans* H99 (Musubire et al., 2018; Osterholzer et al., 2009; Ueno et al., 2019). Importantly, neutrophils display multifaceted activities in the infection of *Cryptococcus.* It has been reported that during neutrophil depletion, γδ T cells exhibit a compensatory increase in IL-17A production, thereby reshaping the Th1/Th17 immune balance. This phenomenon not only suggests that neutrophils are not indispensable during *C. neoformans* infection, but also reveals the complexity of their immunoregulatory functions, warranting further in-depth investigation in this field (Wozniak et al., 2012). Furthermore, during the advanced stages of cryptococcal infection, neutrophils cause pathological damage by the following mechanisms. The cryptococcal GXM inhibits neutrophil extracellular traps formation and TNF-α and IFN-γ production, enhancing cryptococcal immune escape (Brinkmann et al., 2004). Although both *C. neoformans* and *C. gattii* can cross the blood-brain barrier, they exhibit significant differences in their primary target organs and pathogenic mechanisms within the host. Neutrophils facilitate the traversal of *C. neoformans* across the blood-brain barrier, thereby promoting cerebral infection, whereas *C. gattii* primarily induces pulmonary infection (Ngamskulrungroj et al., 2012; Osterholzer et al., 2009). Recent studies have found that LincR-PPP2R5C deficiency attenuates *C. neoformans* infection and increases the bactericidal activity of neutrophils (Yang et al., 2024a). This first discovery greatly enhances our understanding of the regulation of immunity to cryptococcal infection by lncRNA and provides a new pathway in developing cryptococcal immunotherapy.

### 3.4 Monocytes

At the early stage of cryptococcal infection, the CCL2/CCR2 axis recruits classical monocytes (CD14^++^CD16^-^) to lung tissues, where they differentiate into monocyte-derived DCs (MoDCs) and M1 macrophages, thereby promoting an IFN-γ-dominated Th1 immune response (Heung, 2020; Palframan et al., 2001). Studies have demonstrated that PAMPs, such as β-glucan, facilitate the epigenetic reprogramming of monocytes (e.g., H3K4me3 modification), enabling the development of the long-term memory and enhancing the phagocytic and bacteriostatic capabilities of the monocytes during the secondary immune response (Netea et al., 2016). Following the phagocytosis of *C. neoformans*, non-classical CD14^+^CD16^+^ monocytes (Ly6Clow) adhere to the vascular endothelium via VCAM1/VLA4, subsequently penetrating the blood-brain barrier via a Trojan horse’s mechanism (Heung and Hohl, 2019; Sun et al., 2020; J. Xu et al., 2021). The over-recruitment of monocytes to the central nervous system via CCR2 exacerbates neurological damage through TNF-α and IL-1 (Heung and Hohl, 2019; J. Xu et al., 2021; Ziegler-Heitbrock et al., 2010). Notably, a recent study has shown that the reduction of neuronal damage markers and total CD14^+^ monocytes in cerebrospinal fluid improves the outcomes in cryptococcal meningoencephalitis complicated by a postinfectious inflammatory response syndrome (Hargarten et al., 2025). The application of β-glucan and trained immunity agonists as vaccine adjuvants to induce long-lasting anti-cryptococcal memory presents a novel approach for the immunotherapy of cryptococcal infection.

### 3.5 NK cells

NK cells are important effector cells for anti-cryptococcal immunity, controlling *Cryptococcus* by a dual mechanism of IFN-γ immunomodulation and direct killing by perforin (Schmidt et al., 2017). Under the combined stimulation of cytokines such as IL-2, IL-12, IL-15 and IL-18, NK cells are capable of producing IFN-γ and TNF-α, which promote Th1 polarization of CD4^+^ T cells and M1 polarization of macrophages, enhancing systemic immunity against cryptococcal infection (Abel et al., 2018). Human NK cells express the NKp30 receptor, which activates the Src kinase (Fyn/Lyn)/PI3K pathway upon recognizing β-1,3-glucan from *C. neoformans*, promoting perforin polarization toward the fungal contact site and subsequent membrane disruption (Li et al., 2018; Schmidt et al., 2017; Wiseman et al., 2007). Erg5 kinesin regulates the transportation of perforin within NK cells and suppresses the proliferation of *C. neoformans* (Kyei et al., 2016; Ogbomo et al., 2018; Schmidt et al., 2017). Notably, there are differences in the receptor libraries and functions of NK cells between mice and humans (Campuzano and Wormley, 2018). Recent studies have reported that NKG2D is a PRR molecule expressed in lymphocytes but not myeloid cells. NKG2D-deficient mice are significantly more susceptible to *C. neoformans* infection. NKG2D exerts the antifungal effects through the activation of degranulation and killing of NK cells and T cells, which provides a new target for immune intervention against cryptococcal infection (Charpak-Amikam et al., 2024).

### 3.6 γδ T cells

γδ T cells directly disrupt the cell membranes of *Cryptococcus* via perforin and granulysin at the early stage of infection (Nanno et al., 2007; Uezu et al., 2004). The combined use of IL-12 and IL-18 can protect mice from fatal *C. neoformans* infection by inducing NK and γδ T cells to produce IFN-γ and suppressing the production of IL-4 (Qureshi et al., 1999; T. Zhang et al., 1997). Additionally, γδ T cells recruit neutrophils for the early defense via the upregulation of CXCL1/CXCL5 by IL-17A (Wozniak et al., 2012). At the later stage of infection, γδ T cells inhibit the IL-12 production by DCs and the Th1 differentiation through the sustained IL-17A secretion, which attenuates the Th1 response and avoids immune pathological damage (Sato et al., 2020; Uezu et al., 2004). Notably, γδ T cell-dependent TLR2 recognition of sterylglucosides derived from *C. neoformans* is activated without the need for the classical antigen-presentation pathway. γδ T cell deletion completely abolishes the protective effect of the sgl1Δ vaccine and this protective effect depends on the Mincle receptor (Normile et al., 2022; Watanabe et al., 2025). Therefore, sgl1 is a central target gene for cryptococcal vaccine development.

### 3.7 Other innate immune cells

NKT cells activate the immune response by recognizing glycolipid antigens (α-galactosylceramide) presented by CD1d through their unique Vα14-Jα281 TCR. Vα14^+^ NKT cells migrate rapidly to the lungs through the monocyte chemoattractant protein-1-dependent pathway, promoting significantly NK cell-dependent IFN-γ production at the early stage and the differentiation of *C. neofor*mans-specific Th1 cells at the late stage (Kawakami et al., 2001a, 2001b). NKT cell-deficient mice exhibit reduced IFN-γ production, weakened delayed-type hypersensitivity, and delayed clearance of *C. neoformans* in the lungs (Kawakami et al., 2001a). Moreover, the ability of NKT cells against *C. neoformans* depends on the age-related maturity of NKT cells (Blackstock and Murphy, 2004).

Innate lymphoid cells (ILCs) participate in immune regulation during cryptococcal infection. ILC2s activated by IL-33 promote the type 2 immune response by producing IL-4, IL-5, and IL-13, creating a microenvironment conducive to the growth of *C. neoformans* (Elemam et al., 2021; Flaczyk et al., 2013). ILC3s combat the extracellular pathogens by secreting IL-22 and IL-17 (Elemam et al., 2021). Additionally, depletion of inflammatory monocytes, which recruit ILC2s, reduces ILC2 numbers and consequently improves the host’s prognosis (Heung and Hohl, 2019). ILC2s-deficient mice show enhanced Th1 immune responses, increased classically activated macrophages, and improved control of *C. neoformans* infection (Kindermann et al., 2020). Therefore, the reduction in the function and number of ILC2s may enhance the host’s defense against *C. neoformans*.

**Figure 2:**
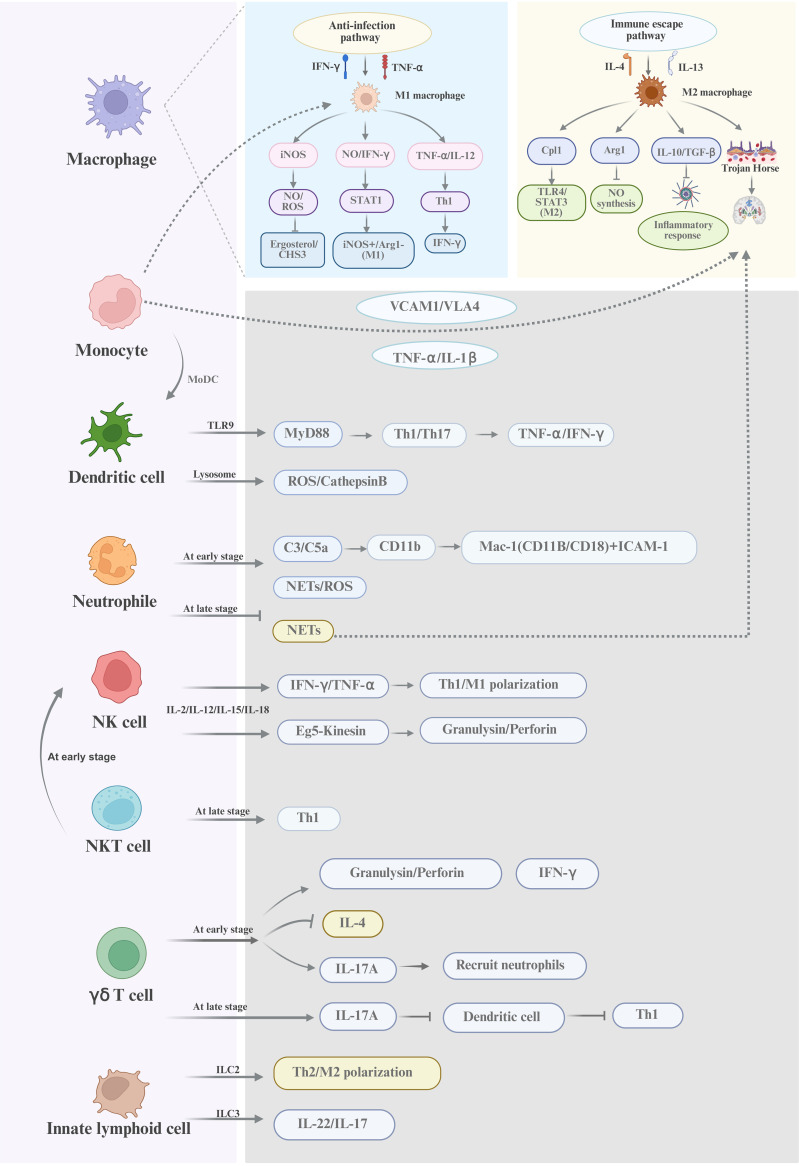
A schematic diagram of the innate immune response against cryptococcal infection Macrophages, dendritic cells, and neutrophils are the innate immune cells with phagocytic functions and play a diversified roles in fighting against *Cryptococcus*. NK cells and NKT cells are non-phagocytic cells and can directly kill *Cryptococcus* through perforin/granulysin-mediated cytolysis or enhance antifungal immunity via IFN-γ production. γδ T cells and innate lymphoid cells exert regulatory functions by modulating Th1/Th17 polarization and the type 2 immune responses. Host defense against *Cryptococcus* involves dynamic interactions among these cellular components, PRRs, soluble mediators, and the pathogen’s immune escape strategies. This multifaceted network underscores the complexity of innate immunity in Cryptococcal infections.

In summary, macrophages, monocytes, dendritic cells, neutrophils, and NK cells are the five innate immune cells that work together through comprehensive mechanisms to fight the infection of *Cryptococcus* (shown in **Figure 2**). Meanwhile, the roles of γδ T cells, NKT cells, and ILCs in cryptococcal infection should be considered. Notably, the bidirectional action mechanisms of the innate immune response to cryptococcal invasion should be taken into consideration in developing the targeted immunotherapy.

## 4 Adaptive immune response

Adaptive immunity plays a critical role in combating cryptococcal infection through providing protection against re-infection. Through the recognition of cryptococcal antigens, adaptive immunity activates immune effector cells, secretes cytokines to eliminate the pathogen, and forms immune memory after the infection, thereby providing protection against future infection (Mukaremera and Nielsen, 2017). Among the various mechanisms, T cell-mediated cellular immunity is fundamental to the adaptive immunity against the infection of *Cryptococcus*. Different types of T lymphocytes are involved in the host’s cellular immune response to *Cryptococcus*. These T cells not only take part in the immune response but also exhibit direct antimicrobial activity against the pathogens. Cellular immunity mediated by T cells is more prominent than humoral immunity mediated by B cells in the defense against *Cryptococcus*. T cells secrete proteins such as granulysin and perforin, which destroy the plasma membrane of *Cryptococcus*, increasing the permeability and ultimately leading to the lysis of the pathogen(Y. Wang et al., 2022).

### 4.1 CD4^+^ T cells

CD4^+^ T cells are commonly classified into four subpopulations, namely Th1, Th2, Th17 and regulatory T cells, which play distinct roles in cryptococcal infection. The subtype of Th1 cells secretes IFN-γ, TNF-α, and IL-2, which exert anti-Cryptoco*ccus* activity (McDermott and Klein, 2018; Oliveira et al., 2025). Among them, IFN-γ not only induces and sustains macrophage polarization towards the M1 type, which is highly effective against pathogens, but also inhibits the differentiation of Th2 cells, thereby blocking the pathological effects induced by IL-4 and IL-13 (Firacative et al., 2018; Leopold Wager et al., 2018). TNF-α promotes the maturation of DCs by enhancing the antigen presentation capabilities, recruits neutrophils to the infection site for direct killing of *C. neoformans*, as well as synergizes with IFN-γ to maintain the M1 polarization of macrophages (Fa et al., 2019). IL-2 activates CD8^+^ T cells and NK cells, facilitating direct killing of *C. neoformans* while ensuring long-lasting T cell immune memory (Levitz and Dupont, 1993). Notably, recent studies have confirmed that Batf3-dependent cDC1 cells are critical for initiating the Th1 response (J. Xu et al., 2024). IL-17 secreted by Th17 cells exhibits a double-edged characteristic in controlling *C. neoformans* infection. At the acute phase, IL-17A directly kills *C. neoformans* by recruiting neutrophils via CXCL1/5, activates the bactericidal functions of macrophages in conjunction with Granulocyte-Macrophage Colony-Stimulating Factor (GM-CSF), and inhibits Th2-mediated mucus secretion and pulmonary fibrosis, thereby providing a protective role. Conversely, during the disseminated phase, Th17 cells compromise the lung barrier, promoting pathogen dissemination, aggravating cerebral edema caused by central nervous system infection, and inhibiting the Th1 immune response, ultimately leading to pathological damage (X. Guo et al., 2022). However, Galectin-3 (Gal-3) inhibits the growth and destabilizes the extracellular vesicle of *C. neoformans* by promoting the Th17 immune response (Almeida et al., 2017). Distinct from Th1 and Th17 cells, Th2 cells produce IL-4, IL-13, IL-5, and IL-10, which facilitate cryptococcal proliferation and tissue damage rather than resisting infection (Elsegeiny et al., 2018; Scriven et al., 2016). However, it is noteworthy that despite the general association of IL-4Rα with Th2-mediated disease progression, during the early stage of infection, the IL-4Rα signaling pathway enhances the host defense through a dual mechanism. On one hand, it upregulates the activity of the IL-12/IFN-γ/NO axis, thereby promoting the Th1 immune response mediated by dendritic cells. On the other hand, it induces the mucus secretion in the airway epithelial cells and enhances the recruitment of CCL2/CCL20-dependent macrophages and dendritic cells (Grahnert et al., 2014). In the later stage, IL-4 and IL-13 induce the polarization of M2 macrophages, while IL-13 induces Th2 cells, mast cells, and basophils to secrete more IL-4, IL-5 and IL-13 through autocrine/paracrine action, which forms a positive feedback loop. Moreover, IL-13 down-regulates the expression of co-stimulatory molecules (such as CD40 and CD80) on DCs, thereby inhibiting their ability to present antigens to CD4^+^ T cells. Simultaneously, it induces the secretion of IL-10, which inhibits the production of IFN-γ by Th1 cells. Furthermore, Th2 cells also activate fibroblasts through IL-13 to upregulate TGF-β and collagen, causing the excessive mucus secretion and airway obstruction, which contributes to the pulmonary fibrosis. Finally, the IL-5-induced infiltration of eosinophils can promote the clearance of pathogens, but excessive activation of eosinophils can lead to tissue damage, such as inflammatory necrosis caused by crystal deposition (Huffnagle et al., 1998; Müller et al., 2007; Ueno et al., 2025). Recent studies have indicated that the IL-33/ST2 axis may amplify lung-resident memory Th2 cells, resulting in persistent type II granulomas (Ueno and Miyazaki, 2023). The shift of Th1 to Th2 is an important vicious element for the reactivation of cryptococcal meningitis in patients with HIV infection (Li et al., 2021; Xu et al., 2019). Additionally, IL-10 secreted by regulatory T cells can indirectly weaken the Th1 immune response by inhibiting the function of antigen-presenting cells, and can also inhibit the excessive inflammatory response mediated by Th2 cells, thereby preventing fatal immune pathological damage (Rubtsov et al., 2008; Schulze et al., 2014). Notably, symptom remission has been correlated with the decrease in the frequencies of activated CD4^+^ and CD8^+^ T cells of patients with cryptococcal meningoencephalitis (Hargarten et al., 2025).

Given the distinct roles of the different CD4^+^ T cell subsets in cryptococcal invasion, a multifaceted approach that promotes the Th1 response, precisely modulates the Th17 response, and inhibits the Th2 response may represent a promising avenue for cryptococcal immunotherapy.

### 4.2 CD8^+^ T cells

CD8^+^ T cells are able to directly damage cryptococcal cell membranes by releasing granulysin and perforin (Okafor and Nielsen, 2024). The killing function of CD8^+^ T cells depends on the activation of CD4^+^ T cells or IL-15, which is defective in patients with HIV infection (Ma et al., 2002; Okafor and Nielsen, 2024; S. Wang et al., 2020). In the absence of CD4^+^ T lymphocytes, CD8^+^ T lymphocytes can still be activated independently and can control the infection by secreting IFN-γ (Lindell et al., 2005). In mouse model, CD8+ T cells are able to prevent the spread of pathogens to the brain during cryptococcal latency. However, in the case of lung infection of *Cryptococcus*, the functions of CD8^+^ T cells may be regulated by CD4^+^ T cells, which affects the IFN-γ production (Okafor and Nielsen, 2024). Immunotherapy needs to be combined with enhancement of CD8^+^ T cell function (vaccines/Chimeric Antigen Receptor T cells(CAR-T cells)/IL-15) and modulation of the balance between Th1 and Th2 responses, with a particular attention to the immune repair and pathological control in HIV patients.

Notably, the most recent report showed that patients with cryptococcal infection but without definitive immunodeficiency retained the ability to produce CD4^+^ and CD8^+^ T cell responses against cryptococcal antigens, which mainly biased towards the Th1 type (high IFN-γ, low IL-4). Moreover, the mouse model further supported that the use of vaccination as a strategy to upgrade the immune responses to prevent clinical *cryptococcus* infection, which provides a basis for the design of cryptococcal vaccines. However, the immune response of peripheral blood mononuclear cells might underestimate the intensity of immune responses at the infection site. Therefore, the vaccine efficacy should be evaluated in combination with tissue samples, such as spleen and lungs (Oliveira et al., 2025).

### 4.3 B cells

The crucial role of B lymphocytes is to mediate humoral immunity against *Cryptococcus* by the production of antibodies (Aslanyan et al., 2017; Boniche et al., 2020). In the deficiency of T lymphocytes, B lymphocytes inhibit the transfer of *C. neoformans* into the brain (Davis and Lionakis, 2018). Immunoglobulin M (IgM) limits the dissemination of *C. neoformans* by restricting the formation of titan cells (Dufaud et al., 2018; Subramaniam et al., 2009; Szymczak et al., 2013; Trevijano-Contador et al., 2020). Immunoglobulin G (IgG) mediates the antibody-dependent cellular cytotoxicity (ADCC) effect of NK cells through FcγR, thereby inhibiting the growth of *C. neoformans* (Nabavi and Murphy, 1986). Immunoglobulin E (IgE) disrupts the immune homeostasis against *C. neoformans* by activating mast cells to release IL-4, thereby inducing a Th2 bias (Casadevall, 2022; Qiu et al., 2013). Research conducted in 2025 demonstrated that immunoglobulin A (IgA) inhibits titan cell formation, which alters the production of *C. neoformans* extracellular vesicles and the expression profiles of metabolic genes (Trevijano-Contador et al., 2025).

**Figure 3:**
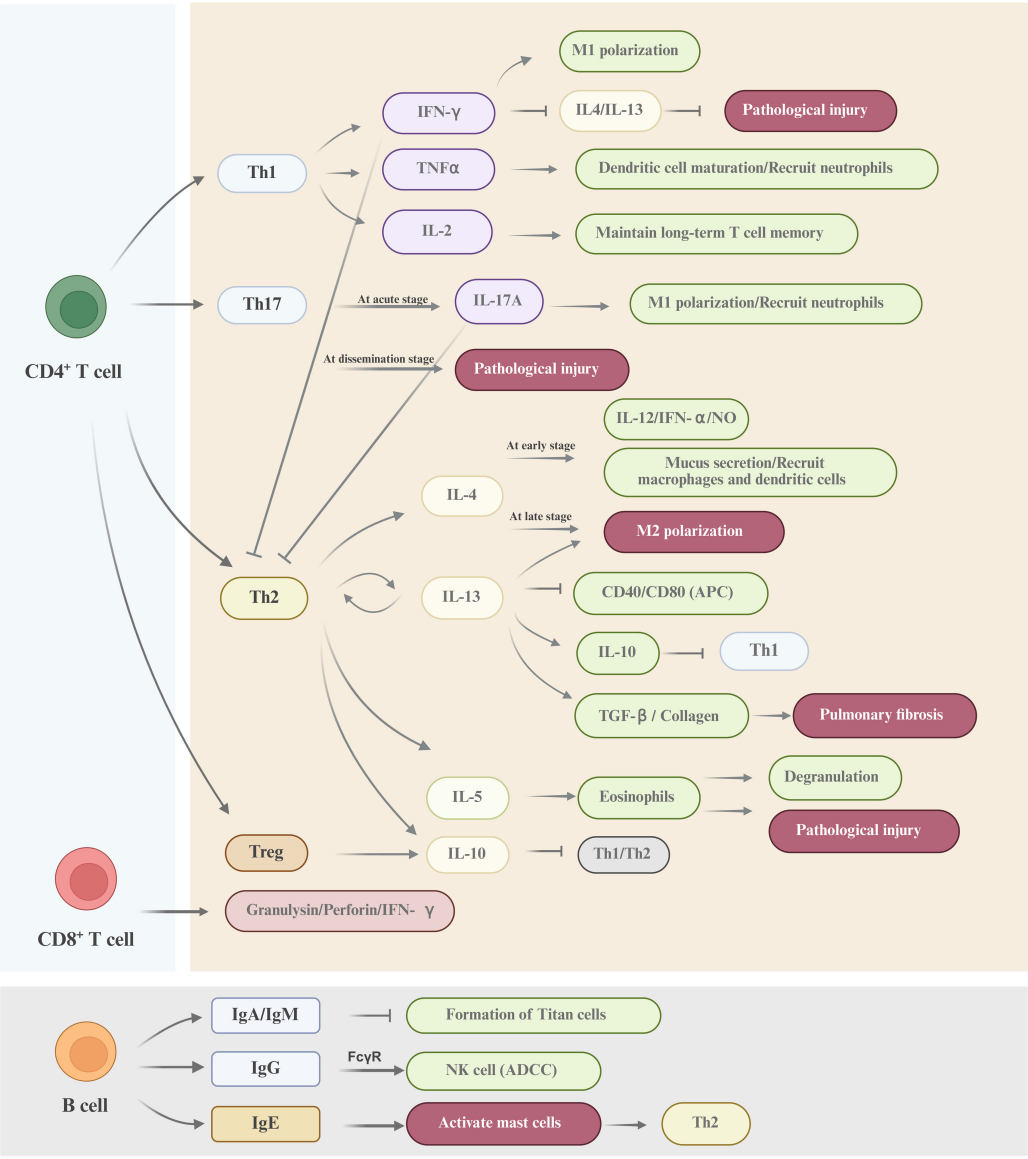
A schematic diagram of the adaptive immune response against cryptococcal infection CD4^+^ T cells, CD8^+^ T cells and B cells constitute the major adaptive immune cell types involved in the defense against *Cryptococcus*. The mechanisms underpinning the adaptive immune response to cryptococcal invasion are highly sophisticated. Collectively, these cells orchestrate a comprehensive defense through synergistic actions, including direct cellular cytotoxicity, cytokine-mediated activation of effector cells, and antibody-dependent mechanisms, to combat *Cryptococcus* infection.

In summary, the adaptive immunity plays a critical role in eliminating the pathogen during cryptococcal infection (shown in **Figure 3**). However, *Cryptococcus* achieves immune evasion and dissemination through mechanisms including capsule shielding, chitin masking of PAMPs, and inducing the differentiation of immunosuppressive cells. The immunotherapy strategies against *Cryptococcus* primarily include: (1) utilizing TLR/Dectin agonists to enhance the phagocytic function of macrophages, (2) inducing Th1/Th17 immune responses through vaccines (such as the Δsgl1 strain), (3) targeting the capsule using CAR-T or monoclonal antibody technology, (4) blocking the IL-4/IL-10 signaling pathway to reverse the immunosuppressive state (Mukaremera and Nielsen, 2017).

## 5 The prospects for immunotherapy

A weakened immune system is a prerequisite for cryptococcal infections, and it is also one of the reasons why traditional antifungal drugs have poor efficacy and are difficult to completely eliminate *Cryptococcus* from the body (Iyer et al., 2021; Liu et al., 2024). Developing new immunotherapies that enhance the host’s anti-*Cryptococcus* immune response has become the key to breaking through the existing treatment barriers (Zhou et al., 2025). The immunotherapeutic methods include monoclonal antibodies (mAbs), cytokines, CAR-T cells, and vaccines, which are focusing on two major directions, targeting pathogen-host interactions and overcoming immune deficiencies (Iyer et al., 2021; Lionakis et al., 2023; Zhou et al., 2025).

There are several mAbs targeting the components of *C. neoformans.* Among them, 18B7 is an mAb for GXM and has completed phase I clinical trials (Larsen et al., 2005). 2G8 is an mAb for β-1,3-glucan (Rachini et al., 2007). DD11 and CC5 are mAbs for chitin oligomers (Figueiredo et al., 2021). Anti-PD-1 mAbs promote the clearance of *C. neoformans* by restoring the activity of Th1/Th17 cells and reducing immunosuppressive cytokines (IL-10/IL-5) (Roussey et al., 2017). Recent studies have shown that mAbs targeting aspartic protease 1 protein significantly increase the survival rate of mice infected with *C. neoformans* (Vernel-Pauillac et al., 2024). Cytokine therapy regulates the immune response to combat *C. neoformans* infection. However, extremely high doses of cytokine, such as IL-12 or IFN-γ, may induce systemic inflammation and aggravate immune reconstitution inflammatory syndrome (Clemons et al., 1994; Jarvis et al., 2014, 2012; Pappas et al., 2004). GM-CSF can activate alveolar macrophages and promote Th1 polarization in the infection of *C. neoformans* (Chen et al., 2016). Notably, the GM-CSF antibody may be a potential risk factor for the infection of *C. gattii*, leading to the increased host’s susceptibility (Yang et al., 2021). However, studies have demonstrated that the nasal administration of GM-CSF can effectively target the lungs and minimize the systemic toxicity in *C. gattii* infection (Hansakon et al., 2024). CAR-T cell therapy modifies T cells through genetic engineering, enabling them to express chimeric antigen receptors targeting capsular polysaccharide GXM, thereby enhancing the ability to clear both *C. neoformans* and *C. gattii* in animal models (Dos Santos et al., 2021). Two types of GXM-specific CARs have been developed, which can recognize multiple types of *Cryptococcus* and significantly activate T cells to secrete IL-2 and upregulate CD69 expression (Dos Santos et al., 2022; MaChado et al., 2023). However, a high level of PD-1 expression is induced, which accelerates the T cell exhaustion. Therefore, the antigen heterogeneity of *Cryptococcus*, the sustained maintenance of T cell activation status, and immune-related adverse reactions are still challenges that need to be overcome (MaChado et al., 2023; Majumder, 2023).The vaccines for *Cryptococcus* include whole-cell vaccine (attenuated live vaccines, inactivated vaccines), subunit vaccine, and mRNA vaccine. The research on cryptococcal vaccines has entered a stage of rapid development with multiple technologies operating in parallel (Avina et al., 2024; Rivera et al., 2022). The design strategies centered on polysaccharide-protein antigens, mRNA-Lipid Nanoparticle (LNP) delivery, and novel adjuvants have demonstrated significant potential (Crawford et al., 2024; Li et al., 2025a, 2025b; Stempinski et al., 2025). Additionally, the novel vaccines based on dendritic cells are developed to enhance the protective immunity against *C. gattii* infection with high pathogenicity in lung (Ueno et al., 2025). In the future, key issues to be addressed include the protective efficacy for immunocompromised hosts, optimization of delivery systems, selection of adjuvants, coverage of multivalent vaccines, and large-scale production in clinical translation (Avina et al., 2024; Rivera et al., 2022).

## 6 Discussion

Cryptococcal immunotherapy is currently at a critical stage of translation from preclinical studies to clinical application. Innovative directions for vaccine development include the following strategies. First, more attention should be paid to the double-edged features of immune cells to avoid immune overactivation. For instance, employing liposome-encapsulated soluble human CD5 to target the alveolar macrophages through nasal administration can locally elevate TNF-α/IFN-γ secretion while avoiding systemic inflammatory storms (Velasco-de Andrés et al., 2020). Furthermore, using pH-sensitive nanoparticles to deliver IFN-γ at the infection site may further mitigate systemic inflammatory responses (Rajesh et al., 2022). Second, multi-omics techniques should be utilized to drive precise antigen design and overcome the limitations of traditional vaccines. Specific approaches involve the identification of conserved virulence factors through phospho-proteomics, the optimization of epitopes using artificial intelligence, and the knockdown of non-protective GXM epitopes (El Arab et al., 2025; Racle et al., 2023; L. Zhang et al., 2021; Zhu et al., 2024). Third, the application of the epigenetic reprogramming mechanism of trained immunity should be highlighted in reshaping innate immune memory. Specifically, the activation of the Dectin-1-Syk signaling axis via β-glucan induces H3K4me3 modification in monocytes and up-regulates the expression of inflammatory vesicle genes, thereby enhancing antifungal activity during secondary infection (Arts et al., 2016; Bekkering et al., 2021; Eastman et al., 2019; Geckin et al., 2022; Netea et al., 2016). Lastly, the mRNA-LNP platform is a promising avenue to accelerate the clinical translation of vaccines (Sandbrink and Shattock, 2020). The synergistic effect of innate immunity and adaptive immune response can be enhanced by incorporating trained immunity adjuvants (e.g., CAF01 or β-glucan), thereby maintaining a high protection rate in immunosuppressed models (Mirza et al., 2017; R. Wang et al., 2024). The first *Cryptococcus* vaccine would be clinically translated as early as possible through the combination of intelligent antigen design, trained immunity memory programming and immune microenvironment remodeling.

## References

[B1] Abel A. M. Yang C. Thakar M. S. Malarkannan S. (2018). Natural killer cells: development, maturation, and clinical utilization. Front. Immunol. 9. doi: 10.3389/fimmu.2018.01869, PMID: 30150991 PMC6099181

[B2] Almeida F. Wolf J. M. da Silva T. A. DeLeon-Rodriguez C. M. Rezende C. P. Pessoni A. M.. (2017). Galectin-3 impacts Cryptococcus neoformans infection through direct antifungal effects. Nat. Commun. 8, 1968. doi: 10.1038/s41467-017-02126-7, PMID: 29213074 PMC5719036

[B3] Arts R. J. W. Carvalho A. La Rocca C. Palma C. Rodrigues F. Silvestre R.. (2016). Immunometabolic pathways in BCG-induced trained immunity. Cell Rep. 17, 2562–2571. doi: 10.1016/j.celrep.2016.11.011, PMID: 27926861 PMC5177620

[B4] Aslanyan L. Ekhar V. V. DeLeon-Rodriguez C. M. Martinez L. R. (2017). Capsular specific IgM enhances complement-mediated phagocytosis and killing of Cryptococcus neoformans by methamphetamine-treated J774.16 macrophage-like cells. Int. Immunopharmacol 49, 77–84. doi: 10.1016/j.intimp.2017.05.024, PMID: 28551495 PMC5599138

[B5] Avina S. L. Pawar S. Rivera A. Xue C. (2024). Will the real immunogens please stand up: exploiting the immunogenic potential of cryptococcal cell antigens in fungal vaccine development. J. Fungi (Basel) 10 (12), 840. doi: 10.3390/jof10120840, PMID: 39728336 PMC11676676

[B6] Bekkering S. Domínguez-Andrés J. Joosten L. A. B. Riksen N. P. Netea M. G. (2021). Trained immunity: reprogramming innate immunity in health and disease. Annu. Rev. Immunol. 39, 667–693. doi: 10.1146/annurev-immunol-102119-073855, PMID: 33637018

[B7] Blackstock R. Murphy J. W. (2004). Age-related resistance of C57BL/6 mice to Cryptococcus neoformans is dependent on maturation of NKT cells. Infect. Immun. 72, 5175–5180. doi: 10.1128/iai.72.9.5175-5180.2004, PMID: 15322012 PMC517463

[B8] Boniche C. Rossi S. A. Kischkel B. Barbalho F. V. Moura Á N. D. Nosanchuk J. D.. (2020). Immunotherapy against systemic fungal infections based on monoclonal antibodies. J. Fungi (Basel) 6 (1), 31. doi: 10.3390/jof6010031, PMID: 32121415 PMC7151209

[B9] Brinkmann V. Reichard U. Goosmann C. Fauler B. Uhlemann Y. Weiss D. S.. (2004). Neutrophil extracellular traps kill bacteria. Science 303, 1532–1535. doi: 10.1126/science.1092385, PMID: 15001782

[B10] Bryan A. M. You J. K. Li G. Kim J. Singh A. Morstein J.. (2021). Cholesterol and sphingomyelin are critical for Fcγ receptor-mediated phagocytosis of Cryptococcus neoformans by macrophages. J. Biol. Chem. 297, 101411. doi: 10.1016/j.jbc.2021.101411, PMID: 34793834 PMC8661020

[B11] Campuzano A. Castro-Lopez N. Martinez A. J. Olszewski M. A. Ganguly A. Leopold Wager C.. (2020). CARD9 is required for classical macrophage activation and the induction of protective immunity against pulmonary cryptococcosis. mBio 11 (1), e03005-19. doi: 10.1128/mBio.03005-19, PMID: 31911495 PMC6946806

[B12] Campuzano A. Wormley F. L. (2018). Innate immunity against cryptococcus, from recognition to elimination. J. Fungi (Basel) 4 (1), 33. doi: 10.3390/jof4010033, PMID: 29518906 PMC5872336

[B13] Casadevall A. (2022). Immunity to invasive fungal diseases. Annu. Rev. Immunol. 40, 121–141. doi: 10.1146/annurev-immunol-101220-034306, PMID: 35007128

[B14] Casalini G. Giacomelli A. Antinori S. (2024). The WHO fungal priority pathogens list: a crucial reappraisal to review the prioritisation. Lancet Microbe 5, 717–724. doi: 10.1016/s2666-5247(24)00042-9, PMID: 38608682

[B15] Chang C. C. Harrison T. S. Bicanic T. A. Chayakulkeeree M. Sorrell T. C. Warris A.. (2024). Global guideline for the diagnosis and management of cryptococcosis: an initiative of the ECMM and ISHAM in cooperation with the ASM. Lancet Infect. Dis. 24, e495–e512. doi: 10.1016/s1473-3099(23)00731-4, PMID: 38346436 PMC11526416

[B16] Charpak-Amikam Y. Kournos M. Kotzur R. Isaacson B. Bagad Brenner T. Gomez-Cesar E.. (2024). The activating receptor NKG2D is an anti-fungal pattern recognition receptor. Nat. Commun. 15, 8664. doi: 10.1038/s41467-024-52913-2, PMID: 39375344 PMC11458907

[B17] Chen G. H. Teitz-Tennenbaum S. Neal L. M. Murdock B. J. Malachowski A. N. Dils A. J.. (2016). Local GM-CSF-dependent differentiation and activation of pulmonary dendritic cells and macrophages protect against progressive cryptococcal lung infection in mice. J. Immunol. 196, 1810–1821. doi: 10.4049/jimmunol.1501512, PMID: 26755822 PMC4744503

[B18] Clemons K. V. Brummer E. Stevens D. A. (1994). Cytokine treatment of central nervous system infection: efficacy of interleukin-12 alone and synergy with conventional antifungal therapy in experimental cryptococcosis. Antimicrob. Agents Chemother. 38, 460–464. doi: 10.1128/aac.38.3.460, PMID: 7911289 PMC284480

[B19] Coelho C. (2024). Batf3-cDC1 control Th1 and fungicidal responses during cryptococcal meningitis: is this enough to control meningitis? mBio 15, e0037524. doi: 10.1128/mbio.00375-24, PMID: 39254303 PMC11481875

[B20] Conn B. N. Wozniak K. L. (2023). Innate pulmonary phagocytes and their interactions with pathogenic cryptococcus species. J. Fungi (Basel) 9 (6), 617. doi: 10.3390/jof9060617, PMID: 37367553 PMC10299524

[B21] Crawford C. J. Liporagi-Lopes L. Coelho C. Santos Junior S. R. Moraes Nicola A. Wear M. P.. (2024). Semisynthetic Glycoconjugate Vaccine Candidates against Cryptococcus neoformans. ACS Infect. Dis. 10, 2089–2100. doi: 10.1021/acsinfecdis.4c00094, PMID: 38819951 PMC11184550

[B22] Dang E. V. Lei S. Radkov A. Volk R. F. Zaro B. W. Madhani H. D. (2022). Secreted fungal virulence effector triggers allergic inflammation via TLR4. Nature 608, 161–167. doi: 10.1038/s41586-022-05005-4, PMID: 35896747 PMC9744105

[B23] da Silva-Junior E. B. Firmino-Cruz L. Guimarães-de-Oliveira J. C. De-Medeiros J. V. R. de Oliveira Nascimento D. Freire-de-Lima M.. (2021). The role of Toll-like receptor 9 in a murine model of Cryptococcus gattii infection. Sci. Rep. 11, 1407. doi: 10.1038/s41598-021-80959-5, PMID: 33446850 PMC7809259

[B24] Davis M. J. Lionakis M. S. (2018). B cells protect T cell-deficient mice from cryptococcal brain invasion. Virulence 9, 25–27. doi: 10.1080/21505594.2017.1393601, PMID: 29125032 PMC7000194

[B25] Davis M. J. Moyer S. Hoke E. S. Sionov E. Mayer-Barber K. D. Barber D. L.. (2019). Pulmonary iron limitation induced by exogenous type I IFN protects mice from cryptococcus gattii independently of T cells. mBio 10 (3), e00799-19. doi: 10.1128/mBio.00799-19, PMID: 31213551 PMC6581853

[B26] Denning D. W. (2024). Global incidence and mortality of severe fungal disease. Lancet Infect. Dis. 24, e428–e438. doi: 10.1016/s1473-3099(23)00692-8, PMID: 38224705

[B27] Diamond R. D. Root R. K. Bennett J. E. (1972). Factors influencing killing of Cryptococcus neoformans by human leukocytes in *vitro* . J. Infect. Dis. 125, 367–376. doi: 10.1093/infdis/125.4.367, PMID: 4553080

[B28] Dos Santos M. H. MaChado M. P. Kumaresan P. R. da Silva T. A. (2021). Titan Cells and Yeast Forms of Cryptococcus neoformans and Cryptococcus gattii Are Recognized by GXMR-CAR. Microorganisms 9 (9), 1886. doi: 10.3390/microorganisms9091886, PMID: 34576780 PMC8467747

[B29] Dos Santos M. H. MaChado M. P. Kumaresan P. R. da Silva T. A. (2022). Modification of hinge/transmembrane and signal transduction domains improves the expression and signaling threshold of GXMR-CAR specific to cryptococcus spp. Cells 11 (21), 3386. doi: 10.3390/cells11213386, PMID: 36359781 PMC9653562

[B30] Dostert C. Pétrilli V. Van Bruggen R. Steele C. Mossman B. T. Tschopp J. (2008). Innate immune activation through Nalp3 inflammasome sensing of asbestos and silica. Science 320, 674–677. doi: 10.1126/science.1156995, PMID: 18403674 PMC2396588

[B31] Dufaud C. Rivera J. Rohatgi S. Pirofski L. A. (2018). Naïve B cells reduce fungal dissemination in Cryptococcus neoformans infected Rag1(-/-) mice. Virulence 9, 173–184. doi: 10.1080/21505594.2017.1370529, PMID: 28837391 PMC5955176

[B32] Eastman A. J. Xu J. Bermik J. Potchen N. den Dekker A. Neal L. M.. (2019). Epigenetic stabilization of DC and DC precursor classical activation by TNFα contributes to protective T cell polarization. Sci. Adv. 5, eaaw9051. doi: 10.1126/sciadv.aaw9051, PMID: 31840058 PMC6892624

[B33] El Arab R. A. Alkhunaizi M. Alhashem Y. N. Al Khatib A. Bubsheet M. Hassanein S. (2025). Artificial intelligence in vaccine research and development: an umbrella review. Front. Immunol. 16. doi: 10.3389/fimmu.2025.1567116, PMID: 40406131 PMC12095282

[B34] Elemam N. M. Ramakrishnan R. K. Hundt J. E. Halwani R. Maghazachi A. A. Hamid Q. (2021). Innate lymphoid cells and natural killer cells in bacterial infections: function, dysregulation, and therapeutic targets. Front. Cell Infect. Microbiol. 11. doi: 10.3389/fcimb.2021.733564, PMID: 34804991 PMC8602108

[B35] Elsegeiny W. Marr K. A. Williamson P. R. (2018). Immunology of cryptococcal infections: developing a rational approach to patient therapy. Front. Immunol. 9. doi: 10.3389/fimmu.2018.00651, PMID: 29670625 PMC5893745

[B36] Fa Z. Xu J. Yi J. Sang J. Pan W. Xie Q.. (2019). TNF-α-producing cryptococcus neoformans exerts protective effects on host defenses in murine pulmonary cryptococcosis. Front. Immunol. 10. doi: 10.3389/fimmu.2019.01725, PMID: 31404168 PMC6677034

[B37] Figueiredo A. B. C. Fonseca F. L. Kuczera D. Conte F. P. Arissawa M. Rodrigues M. L. (2021). Monoclonal antibodies against cell wall chitooligomers as accessory tools for the control of cryptococcosis. Antimicrob. Agents Chemother. 65, e0118121. doi: 10.1128/aac.01181-21, PMID: 34570650 PMC8597760

[B38] Filippi M. D. (2019). Neutrophil transendothelial migration: updates and new perspectives. Blood 133, 2149–2158. doi: 10.1182/blood-2018-12-844605, PMID: 30898863 PMC6524565

[B39] Firacative C. Gressler A. E. Schubert K. Schulze B. Müller U. Brombacher F.. (2018). Identification of T helper (Th)1- and Th2-associated antigens of Cryptococcus neoformans in a murine model of pulmonary infection. Sci. Rep. 8, 2681. doi: 10.1038/s41598-018-21039-z, PMID: 29422616 PMC5805727

[B40] Flaczyk A. Duerr C. U. Shourian M. Lafferty E. I. Fritz J. H. Qureshi S. T. (2013). IL-33 signaling regulates innate and adaptive immunity to Cryptococcus neoformans. J. Immunol. 191, 2503–2513. doi: 10.4049/jimmunol.1300426, PMID: 23894196

[B41] Franchi L. Muñoz-Planillo R. Núñez G. (2012). Sensing and reacting to microbes through the inflammasomes. Nat. Immunol. 13, 325–332. doi: 10.1038/ni.2231, PMID: 22430785 PMC3449002

[B42] Francis V. I. Liddle C. Camacho E. Kulkarni M. Junior S. R. S. Harvey J. A.. (2024). Cryptococcus neoformans rapidly invades the murine brain by sequential breaching of airway and endothelial tissues barriers, followed by engulfment by microglia. mBio 15, e0307823. doi: 10.1128/mbio.03078-23, PMID: 38511961 PMC11005363

[B43] Garcia-Rubio R. de Oliveira H. C. Rivera J. Trevijano-Contador N. (2019). The fungal cell wall: candida, cryptococcus, and aspergillus species. Front. Microbiol. 10. doi: 10.3389/fmicb.2019.02993, PMID: 31993032 PMC6962315

[B44] Geckin B. Konstantin Föhse F. Domínguez-Andrés J. Netea M. G. (2022). Trained immunity: implications for vaccination. Curr. Opin. Immunol. 77, 102190. doi: 10.1016/j.coi.2022.102190, PMID: 35597182

[B45] Giles S. S. Dagenais T. R. Botts M. R. Keller N. P. Hull C. M. (2009). Elucidating the pathogenesis of spores from the human fungal pathogen Cryptococcus neoformans. Infect. Immun. 77, 3491–3500. doi: 10.1128/iai.00334-09, PMID: 19451235 PMC2715683

[B46] Goughenour K. D. Nair A. S. Xu J. Olszewski M. A. Wozniak K. L. (2023). Dendritic cells: multifunctional roles in host defenses to cryptococcus infections. J. Fungi (Basel) 9 (11), 1050. doi: 10.3390/jof9111050, PMID: 37998856 PMC10672120

[B47] Gow N. A. R. Lenardon M. D. (2023). Architecture of the dynamic fungal cell wall. Nat. Rev. Microbiol. 21, 248–259. doi: 10.1038/s41579-022-00796-9, PMID: 36266346

[B48] Grahnert A. Richter T. Piehler D. Eschke M. Schulze B. Müller U.. (2014). IL-4 receptor-alpha-dependent control of Cryptococcus neoformans in the early phase of pulmonary infection. PloS One 9, e87341. doi: 10.1371/journal.pone.0087341, PMID: 24475277 PMC3903725

[B49] Gross O. Gewies A. Finger K. Schäfer M. Sparwasser T. Peschel C.. (2006). Card9 controls a non-TLR signalling pathway for innate anti-fungal immunity. Nature 442, 651–656. doi: 10.1038/nature04926, PMID: 16862125

[B50] Guo C. Chen M. Fa Z. Lu A. Fang W. Sun B.. (2014). Acapsular Cryptococcus neoformans activates the NLRP3 inflammasome. Microbes Infect. 16, 845–854. doi: 10.1016/j.micinf.2014.08.013, PMID: 25193031

[B51] Guo X. Mao X. Tian D. Liao Y. Su B. Ye C.. (2022). Cryptococcus neoformans infection induces IL-17 production by promoting STAT3 phosphorylation in CD4(+) T cells. Front. Immunol. 13. doi: 10.3389/fimmu.2022.872286, PMID: 35720334 PMC9197778

[B52] Hagen F. Khayhan K. Theelen B. Kolecka A. Polacheck I. Sionov E.. (2015). Recognition of seven species in the Cryptococcus gattii/Cryptococcus neoformans species complex. Fungal Genet. Biol. 78, 16–48. doi: 10.1016/j.fgb.2015.02.009, PMID: 25721988

[B53] Hansakon A. Khampoongern R. Schiller L. Jeerawattanawart S. Angkasekwinai P. (2024). Effect of intranasal administration of Granulocyte-Macrophage Colony-Stimulating Factor on pulmonary Cryptococcus gattii infection. Int. Immunopharmacol 142, 113259. doi: 10.1016/j.intimp.2024.113259, PMID: 39332096

[B54] Hansakon A. Ngamphiw C. Tongsima S. Angkasekwinai P. (2023). Arginase 1 Expression by Macrophages Promotes Cryptococcus neoformans Proliferation and Invasion into Brain Microvascular Endothelial Cells. J. Immunol. 210, 408–419. doi: 10.4049/jimmunol.2200592, PMID: 36548474

[B55] Hargarten J. C. Ssebambulidde K. Anjum S. H. Vaughan M. J. Xu J. Ganguly A.. (2025). Pathway-instructed therapeutic selection of ruxolitinib reduces neuroinflammation in fungal postinfectious inflammatory syndrome. Sci. Adv. 11, eadi9885. doi: 10.1126/sciadv.adi9885, PMID: 40117367 PMC11927619

[B56] Heung L. J. (2017). Innate immune responses to cryptococcus. J. Fungi (Basel) 3 (3), 35. doi: 10.3390/jof3030035, PMID: 28936464 PMC5604851

[B57] Heung L. J. (2020). Monocytes and the host response to fungal pathogens. Front. Cell Infect. Microbiol. 10. doi: 10.3389/fcimb.2020.00034, PMID: 32117808 PMC7031161

[B58] Heung L. J. Hohl T. M. (2019). Inflammatory monocytes are detrimental to the host immune response during acute infection with Cryptococcus neoformans. PloS Pathog. 15, e1007627. doi: 10.1371/journal.ppat.1007627, PMID: 30897162 PMC6428256

[B59] Hole C. R. Wager C. M. L. Castro-Lopez N. Campuzano A. Cai H. Wozniak K. L.. (2019). Induction of memory-like dendritic cell responses in *vivo* . Nat. Commun. 10, 2955. doi: 10.1038/s41467-019-10486-5, PMID: 31273203 PMC6609631

[B60] Huang H. R. Li F. Han H. Xu X. Li N. Wang S.. (2018). Dectin-3 Recognizes Glucuronoxylomannan of Cryptococcus neoformans Serotype AD and Cryptococcus gattii Serotype B to Initiate Host Defense Against Cryptococcosis. Front. Immunol. 9. doi: 10.3389/fimmu.2018.01781, PMID: 30131805 PMC6090260

[B61] Huffnagle G. B. Boyd M. B. Street N. E. Lipscomb M. F. (1998). IL-5 is required for eosinophil recruitment, crystal deposition, and mononuclear cell recruitment during a pulmonary Cryptococcus neoformans infection in genetically susceptible mice (C57BL/6). J. Immunol. 160, 2393–2400. doi: 10.4049/jimmunol.160.5.2393, PMID: 9498782

[B62] Huston S. M. Li S. S. Stack D. Timm-McCann M. Jones G. J. Islam A.. (2013). Cryptococcus gattii is killed by dendritic cells, but evades adaptive immunity by failing to induce dendritic cell maturation. J. Immunol. 191, 249–261. doi: 10.4049/jimmunol.1202707, PMID: 23740956

[B63] Iyer K. R. Revie N. M. Fu C. Robbins N. Cowen L. E. (2021). Treatment strategies for cryptococcal infection: challenges, advances and future outlook. Nat. Rev. Microbiol. 19, 454–466. doi: 10.1038/s41579-021-00511-0, PMID: 33558691 PMC7868659

[B64] Jamil K. Polyak M. J. Feehan D. D. Surmanowicz P. Stack D. Li S. S.. (2020). Phagosomal F-actin retention by cryptococcus gattii induces dendritic cell immunoparalysis. mBio 11 (6), e01821-20. doi: 10.1128/mBio.01821-20, PMID: 33234684 PMC7701985

[B65] Jarvis J. N. Bicanic T. Loyse A. Namarika D. Jackson A. Nussbaum J. C.. (2014). Determinants of mortality in a combined cohort of 501 patients with HIV-associated Cryptococcal meningitis: implications for improving outcomes. Clin. Infect. Dis. 58, 736–745. doi: 10.1093/cid/cit794, PMID: 24319084 PMC3922213

[B66] Jarvis J. N. Meintjes G. Rebe K. Williams G. N. Bicanic T. Williams A.. (2012). Adjunctive interferon-γ immunotherapy for the treatment of HIV-associated cryptococcal meningitis: a randomized controlled trial. Aids 26, 1105–1113. doi: 10.1097/QAD.0b013e3283536a93, PMID: 22421244 PMC3640254

[B67] Kawakami K. Kinjo Y. Uezu K. Yara S. Miyagi K. Koguchi Y.. (2001a). Monocyte chemoattractant protein-1-dependent increase of V alpha 14 NKT cells in lungs and their roles in Th1 response and host defense in cryptococcal infection. J. Immunol. 167, 6525–6532. doi: 10.4049/jimmunol.167.11.6525, PMID: 11714821

[B68] Kawakami K. Kinjo Y. Yara S. Koguchi Y. Uezu K. Nakayama T.. (2001b). Activation of Valpha14(+) natural killer T cells by alpha-galactosylceramide results in development of Th1 response and local host resistance in mice infected with Cryptococcus neoformans. Infect. Immun. 69, 213–220. doi: 10.1128/iai.69.1.213-220.2001, PMID: 11119508 PMC97874

[B69] Kindermann M. Knipfer L. Obermeyer S. Müller U. Alber G. Bogdan C.. (2020). Group 2 innate lymphoid cells (ILC2) suppress beneficial type 1 immune responses during pulmonary cryptococcosis. Front. Immunol. 11. doi: 10.3389/fimmu.2020.00209, PMID: 32117319 PMC7034304

[B70] Kitai Y. Sato K. Tanno D. Yuan X. Umeki A. Kasamatsu J.. (2021). Role of dectin-2 in the phagocytosis of cryptococcus neoformans by dendritic cells. Infect. Immun. 89, e0033021. doi: 10.1128/iai.00330-21, PMID: 34251289 PMC8445189

[B71] Kyei S. K. Ogbomo H. Li S. Timm-McCann M. Xiang R. F. Huston S. M.. (2016). Mechanisms by which interleukin-12 corrects defective NK cell anticryptococcal activity in HIV-infected patients. mBio 7 (4), e00878-16. doi: 10.1128/mBio.00878-16, PMID: 27555306 PMC4999542

[B72] Larsen R. A. Pappas P. G. Perfect J. Aberg J. A. Casadevall A. Cloud G. A.. (2005). Phase I evaluation of the safety and pharmacokinetics of murine-derived anticryptococcal antibody 18B7 in subjects with treated cryptococcal meningitis. Antimicrob. Agents Chemother. 49, 952–958. doi: 10.1128/aac.49.3.952-958.2005, PMID: 15728888 PMC549259

[B73] Lei G. Chen M. Li H. Niu J. L. Wu S. Mao L.. (2013). Biofilm from a clinical strain of Cryptococcus neoformans activates the NLRP3 inflammasome. Cell Res. 23, 965–968. doi: 10.1038/cr.2013.49, PMID: 23567555 PMC3698630

[B74] Leopold Wager C. M. Hole C. R. Campuzano A. Castro-Lopez N. Cai H. Caballero Van Dyke M. C.. (2018). IFN-γ immune priming of macrophages *in vivo* induces prolonged STAT1 binding and protection against Cryptococcus neoformans. PloS Pathog. 14, e1007358. doi: 10.1371/journal.ppat.1007358, PMID: 30304063 PMC6197699

[B75] Leopold Wager C. M. Wormley F. L. Jr. (2014). Classical versus alternative macrophage activation: the Ying and the Yang in host defense against pulmonary fungal infections. Mucosal Immunol. 7, 1023–1035. doi: 10.1038/mi.2014.65, PMID: 25073676

[B76] Levitz S. M. Dupont M. P. (1993). Phenotypic and functional characterization of human lymphocytes activated by interleukin-2 to directly inhibit growth of Cryptococcus neoformans in *vitro* . J. Clin. Invest. 91, 1490–1498. doi: 10.1172/jci116354, PMID: 7682573 PMC288124

[B77] Li Y. Ambati S. Meagher R. B. Lin X. (2025a). Developing mRNA lipid nanoparticle vaccine effective for cryptococcosis in a murine model. NPJ Vaccines 10, 24. doi: 10.1038/s41541-025-01079-z, PMID: 39905025 PMC11794474

[B78] Li Z. Lu G. Meng G. (2019). Pathogenic fungal infection in the lung. Front. Immunol. 10. doi: 10.3389/fimmu.2019.01524, PMID: 31333658 PMC6616198

[B79] Li S. S. Ogbomo H. Mansour M. K. Xiang R. F. Szabo L. Munro F.. (2018). Identification of the fungal ligand triggering cytotoxic PRR-mediated NK cell killing of Cryptococcus and Candida. Nat. Commun. 9, 751. doi: 10.1038/s41467-018-03014-4, PMID: 29467448 PMC5821813

[B80] Li Y. Pham T. Hipsher K. Lee C. W. J. Jiao J. Penninger J. M.. (2025b). Identification of a protective antigen reveals the trade-off between iron acquisition and antigen exposure in a global fungal pathogen. Proc. Natl. Acad. Sci. U.S.A. 122, e2420898122. doi: 10.1073/pnas.2420898122, PMID: 39946532 PMC11848283

[B81] Li A. Zhu W. Yin J. Huang X. Sun L. Hua W.. (2021). A preliminary study on the characteristics of Th1/Th2 immune response in cerebrospinal fluid of AIDS patients with cryptococcal meningitis. BMC Infect. Dis. 21, 500. doi: 10.1186/s12879-021-06138-z, PMID: 34051748 PMC8164222

[B82] Lindell D. M. Moore T. A. McDonald R. A. Toews G. B. Huffnagle G. B. (2005). Generation of antifungal effector CD8+ T cells in the absence of CD4+ T cells during Cryptococcus neoformans infection. J. Immunol. 174, 7920–7928. doi: 10.4049/jimmunol.174.12.7920, PMID: 15944298

[B83] Lionakis M. S. Drummond R. A. Hohl T. M. (2023). Immune responses to human fungal pathogens and therapeutic prospects. Nat. Rev. Immunol. 23, 433–452. doi: 10.1038/s41577-022-00826-w, PMID: 36600071 PMC9812358

[B84] Liu W. Gao Y. Ding C. (2024). Exploring emerging drug responses in Cryptococcus. Trends Microbiol. 32, 940–943. doi: 10.1016/j.tim.2024.07.002, PMID: 39033069

[B85] Ma L. L. Spurrell J. C. Wang J. F. Neely G. G. Epelman S. Krensky A. M.. (2002). CD8 T cell-mediated killing of Cryptococcus neoformans requires granulysin and is dependent on CD4 T cells and IL-15. J. Immunol. 169, 5787–5795. doi: 10.4049/jimmunol.169.10.5787, PMID: 12421959

[B86] MaChado M. P. Dos Santos M. H. Guimarães J. G. de Campos G. Y. Oliveira Brito P. K. M. Ferreira C. M. G.. (2023). GXMR-CAR containing distinct GXM-specific single-chain variable fragment (scFv) mediated the cell activation against Cryptococcus spp. And had difference in the strength of tonic signaling. Bioengineered 14, 2281059. doi: 10.1080/21655979.2023.2281059, PMID: 37978838 PMC10761124

[B87] Majumder A. (2023). Evolving CAR-T-cell therapy for cancer treatment: from scientific discovery to cures. Cancers (Basel) 16 (1), 39. doi: 10.3390/cancers16010039, PMID: 38201467 PMC10777914

[B88] Marina C. L. de Castro R. J. A. Bellozi P. Cruz A. M. Bürgel P. H. Potter P. G. W.. (2025). Immunometabolic reprogramming in macrophages infected with active and dormant Cryptococcus neoformans: differential modulation of respiration, glycolysis, and fatty acid utilization. Infect. Immun. 93, e0048724. doi: 10.1128/iai.00487-24, PMID: 39714095 PMC11834436

[B89] May R. C. Stone N. R. Wiesner D. L. Bicanic T. Nielsen K. (2016). Cryptococcus: from environmental saprophyte to global pathogen. Nat. Rev. Microbiol. 14, 106–117. doi: 10.1038/nrmicro.2015.6, PMID: 26685750 PMC5019959

[B90] McDermott A. J. Klein B. S. (2018). Helper T-cell responses and pulmonary fungal infections. Immunology 155, 155–163. doi: 10.1111/imm.12953, PMID: 29781185 PMC6142286

[B91] McGreal E. P. Rosas M. Brown G. D. Zamze S. Wong S. Y. Gordon S.. (2006). The carbohydrate-recognition domain of Dectin-2 is a C-type lectin with specificity for high mannose. Glycobiology 16, 422–430. doi: 10.1093/glycob/cwj077, PMID: 16423983

[B92] Means T. K. Mylonakis E. Tampakakis E. Colvin R. A. Seung E. Puckett L.. (2009). Evolutionarily conserved recognition and innate immunity to fungal pathogens by the scavenger receptors SCARF1 and CD36. J. Exp. Med. 206, 637–653. doi: 10.1084/jem.20082109, PMID: 19237602 PMC2699123

[B93] Mirza Z. Soto E. R. Dikengil F. Levitz S. M. Ostroff G. R. (2017). Beta-glucan particles as vaccine adjuvant carriers. Methods Mol. Biol. 1625, 143–157. doi: 10.1007/978-1-4939-7104-6_11, PMID: 28584989

[B94] Mukaremera L. Nielsen K. (2017). Adaptive immunity to cryptococcus neoformans infections. J. Fungi (Basel) 3 (4), 64. doi: 10.3390/jof3040064, PMID: 29333430 PMC5753166

[B95] Müller U. Stenzel W. Köhler G. Werner C. Polte T. Hansen G.. (2007). IL-13 induces disease-promoting type 2 cytokines, alternatively activated macrophages and allergic inflammation during pulmonary infection of mice with Cryptococcus neoformans. J. Immunol. 179, 5367–5377. doi: 10.4049/jimmunol.179.8.5367, PMID: 17911623

[B96] Musubire A. K. Meya D. B. Rhein J. Meintjes G. Bohjanen P. R. Nuwagira E.. (2018). Blood neutrophil counts in HIV-infected patients with cryptococcal meningitis: Association with mortality. PloS One 13, e0209337. doi: 10.1371/journal.pone.0209337, PMID: 30596708 PMC6312212

[B97] Nabavi N. Murphy J. W. (1986). Antibody-dependent natural killer cell-mediated growth inhibition of Cryptococcus neoformans. Infect. Immun. 51, 556–562. doi: 10.1128/iai.51.2.556-562.1986, PMID: 3510982 PMC262375

[B98] Nakamura K. Kinjo T. Saijo S. Miyazato A. Adachi Y. Ohno N.. (2007). Dectin-1 is not required for the host defense to Cryptococcus neoformans. Microbiol. Immunol. 51, 1115–1119. doi: 10.1111/j.1348-0421.2007.tb04007.x, PMID: 18037789

[B99] Nakamura K. Miyazato A. Xiao G. Hatta M. Inden K. Aoyagi T.. (2008). Deoxynucleic acids from Cryptococcus neoformans activate myeloid dendritic cells via a TLR9-dependent pathway. J. Immunol. 180, 4067–4074. doi: 10.4049/jimmunol.180.6.4067, PMID: 18322216

[B100] Nakamura Y. Sato K. Yamamoto H. Matsumura K. Matsumoto I. Nomura T.. (2015). Dectin-2 deficiency promotes Th2 response and mucin production in the lungs after pulmonary infection with Cryptococcus neoformans. Infect. Immun. 83, 671–681. doi: 10.1128/iai.02835-14, PMID: 25422263 PMC4294247

[B101] Nanno M. Shiohara T. Yamamoto H. Kawakami K. Ishikawa H. (2007). gammadelta T cells: firefighters or fire boosters in the front lines of inflammatory responses. Immunol. Rev. 215, 103–113. doi: 10.1111/j.1600-065X.2006.00474.x, PMID: 17291282

[B102] Netea M. G. Joosten L. A. Latz E. Mills K. H. Natoli G. Stunnenberg H. G.. (2016). Trained immunity: A program of innate immune memory in health and disease. Science 352, aaf1098. doi: 10.1126/science.aaf1098, PMID: 27102489 PMC5087274

[B103] Ngamskulrungroj P. Chang Y. Sionov E. Kwon-Chung K. J. (2012). The primary target organ of Cryptococcus gattii is different from that of Cryptococcus neoformans in a murine model. mBio 3 (3), e00103-12. doi: 10.1128/mBio.00103-12, PMID: 22570277 PMC3350374

[B104] Normile T. G. Chu T. H. Sheridan B. S. Del Poeta M. (2022). Vaccine protection by Cryptococcus neoformans Δsgl1 is mediated by γδ T cells via TLR2 signaling. Mucosal Immunol. 15, 1416–1430. doi: 10.1038/s41385-022-00570-3, PMID: 36229573 PMC9705245

[B105] Ogbomo H. Timm-McCann M. Barnes T. Xiang R. F. Jamil K. Ganguly A.. (2018). Granule-dependent NK cell killing of cryptococcus requires kinesin to reposition the cytolytic machinery for directed cytotoxicity. Cell Rep. 24, 3017–3032. doi: 10.1016/j.celrep.2018.08.027, PMID: 30208325

[B106] Okafor E. C. Nielsen K. (2024). State of the Field: Cytotoxic Immune Cell Responses in C. neoformans and C. deneoformans Infection. J. Fungi (Basel) 10 (10), 712. doi: 10.3390/jof10100712, PMID: 39452664 PMC11508571

[B107] Oliveira L. V. N. Hargarten J. C. Wang R. Carlson D. Park Y. D. Specht C. A.. (2025). Peripheral blood CD4(+) and CD8(+) T cell responses to Cryptococcus candidate vaccine antigens in human subjects with and without cryptococcosis. J. Infect. 91, 106521. doi: 10.1016/j.jinf.2025.106521, PMID: 40449806 PMC12212068

[B108] Onyishi C. U. Desanti G. E. Wilkinson A. L. Lara-Reyna S. Frickel E. M. Fejer G.. (2023). Toll-like receptor 4 and macrophage scavenger receptor 1 crosstalk regulates phagocytosis of a fungal pathogen. Nat. Commun. 14, 4895. doi: 10.1038/s41467-023-40635-w, PMID: 37580395 PMC10425417

[B109] Onyishi C. U. Jeon Y. Fejer G. Mukhopadhyay S. Gordon S. May R. C. (2024). Loss of the scavenger receptor MARCO results in uncontrolled vomocytosis of fungi from macrophages. Eur. J. Immunol. 54, e2350771. doi: 10.1002/eji.202350771, PMID: 38494423

[B110] Osterholzer J. J. Milam J. E. Chen G. H. Toews G. B. Huffnagle G. B. Olszewski M. A. (2009). Role of dendritic cells and alveolar macrophages in regulating early host defense against pulmonary infection with Cryptococcus neoformans. Infect. Immun. 77, 3749–3758. doi: 10.1128/iai.00454-09, PMID: 19564388 PMC2737986

[B111] Palframan R. T. Jung S. Cheng G. Weninger W. Luo Y. Dorf M.. (2001). Inflammatory chemokine transport and presentation in HEV: a remote control mechanism for monocyte recruitment to lymph nodes in inflamed tissues. J. Exp. Med. 194, 1361–1373. doi: 10.1084/jem.194.9.1361, PMID: 11696600 PMC2195988

[B112] Pappas P. G. Bustamante B. Ticona E. Hamill R. J. Johnson P. C. Reboli A.. (2004). Recombinant interferon- gamma 1b as adjunctive therapy for AIDS-related acute cryptococcal meningitis. J. Infect. Dis. 189, 2185–2191. doi: 10.1086/420829, PMID: 15181565

[B113] Pathakumari B. Liang G. Liu W. (2020). Immune defence to invasive fungal infections: A comprehensive review. BioMed. Pharmacother. 130, 110550. doi: 10.1016/j.biopha.2020.110550, PMID: 32739740

[B114] Pétrilli V. Papin S. Dostert C. Mayor A. Martinon F. Tschopp J. (2007). Activation of the NALP3 inflammasome is triggered by low intracellular potassium concentration. Cell Death Differ 14, 1583–1589. doi: 10.1038/sj.cdd.4402195, PMID: 17599094

[B115] Qiu Y. Dayrit J. K. Davis M. J. Carolan J. F. Osterholzer J. J. Curtis J. L.. (2013). Scavenger receptor A modulates the immune response to pulmonary Cryptococcus neoformans infection. J. Immunol. 191, 238–248. doi: 10.4049/jimmunol.1203435, PMID: 23733871 PMC4007509

[B116] Qureshi M. H. Zhang T. Koguchi Y. Nakashima K. Okamura H. Kurimoto M.. (1999). Combined effects of IL-12 and IL-18 on the clinical course and local cytokine production in murine pulmonary infection with Cryptococcus neoformans. Eur. J. Immunol. 29, 643–649. doi: 10.1002/(sici)1521-4141(199902)29:02<643::Aid-immu643>3.0.Co;2-e, PMID: 10064081

[B117] Rachini A. Pietrella D. Lupo P. Torosantucci A. Chiani P. Bromuro C.. (2007). An anti-beta-glucan monoclonal antibody inhibits growth and capsule formation of Cryptococcus neoformans *in vitro* and exerts therapeutic, anticryptococcal activity *in vivo* . Infect. Immun. 75, 5085–5094. doi: 10.1128/iai.00278-07, PMID: 17606600 PMC2168274

[B118] Racle J. Guillaume P. Schmidt J. Michaux J. Larabi A. Lau K.. (2023). Machine learning predictions of MHC-II specificities reveal alternative binding mode of class II epitopes. Immunity 56, 1359–1375.e1313. doi: 10.1016/j.immuni.2023.03.009, PMID: 37023751

[B119] Rajasingham R. Govender N. P. Jordan A. Loyse A. Shroufi A. Denning D. W.. (2022). The global burden of HIV-associated cryptococcal infection in adults in 2020: a modelling analysis. Lancet Infect. Dis. 22, 1748–1755. doi: 10.1016/s1473-3099(22)00499-6, PMID: 36049486 PMC9701154

[B120] Rajesh S. Gangadoo S. Nguyen H. Zhai J. Dekiwadia C. Drummond C. J.. (2022). Application of fluconazole-loaded pH-sensitive lipid nanoparticles for enhanced antifungal therapy. ACS Appl. Mater Interfaces. 14 (29), 32845–32854. doi: 10.1021/acsami.2c05165, PMID: 35850116

[B121] Ramirez-Ortiz Z. G. Means T. K. (2012). The role of dendritic cells in the innate recognition of pathogenic fungi (A. fumigatus, C. neoformans and C. albicans). Virulence 3, 635–646. doi: 10.4161/viru.22295, PMID: 23076328 PMC3545945

[B122] Reese A. J. Doering T. L. (2003). Cell wall alpha-1,3-glucan is required to anchor the Cryptococcus neoformans capsule. Mol. Microbiol. 50, 1401–1409. doi: 10.1046/j.1365-2958.2003.03780.x, PMID: 14622425

[B123] Rella A. Mor V. Farnoud A. M. Singh A. Shamseddine A. A. Ivanova E.. (2015). Role of Sterylglucosidase 1 (Sgl1) on the pathogenicity of Cryptococcus neoformans: potential applications for vaccine development. Front. Microbiol. 6. doi: 10.3389/fmicb.2015.00836, PMID: 26322039 PMC4531891

[B124] Rivera A. Lodge J. Xue C. (2022). Harnessing the immune response to fungal pathogens for vaccine development. Annu. Rev. Microbiol. 76, 703–726. doi: 10.1146/annurev-micro-041020-111511, PMID: 35759871 PMC11926770

[B125] Robbins N. Caplan T. Cowen L. E. (2017). Molecular evolution of antifungal drug resistance. Annu. Rev. Microbiol. 71, 753–775. doi: 10.1146/annurev-micro-030117-020345, PMID: 28886681

[B126] Roussey J. A. Viglianti S. P. Teitz-Tennenbaum S. Olszewski M. A. Osterholzer J. J. (2017). Anti-PD-1 antibody treatment promotes clearance of persistent cryptococcal lung infection in mice. J. Immunol. 199, 3535–3546. doi: 10.4049/jimmunol.1700840, PMID: 29038249 PMC5687305

[B127] Rubtsov Y. P. Rasmussen J. P. Chi E. Y. Fontenot J. Castelli L. Ye X.. (2008). Regulatory T cell-derived interleukin-10 limits inflammation at environmental interfaces. Immunity 28, 546–558. doi: 10.1016/j.immuni.2008.02.017, PMID: 18387831

[B128] Saijo S. Iwakura Y. (2011). Dectin-1 and Dectin-2 in innate immunity against fungi. Int. Immunol. 23, 467–472. doi: 10.1093/intimm/dxr046, PMID: 21677049

[B129] Sandbrink J. B. Shattock R. J. (2020). RNA vaccines: A suitable platform for tackling emerging pandemics? Front. Immunol. 11. doi: 10.3389/fimmu.2020.608460, PMID: 33414790 PMC7783390

[B130] Sato Y. Sato K. Yamamoto H. Kasamatsu J. Miyasaka T. Tanno D.. (2020). Limited Role of Mincle in the Host Defense against Infection with Cryptococcus deneoformans. Infect. Immun. 88 (11), e00400-20. doi: 10.1128/iai.00400-20, PMID: 32868343 PMC7573449

[B131] Sato K. Yamamoto H. Nomura T. Kasamatsu J. Miyasaka T. Tanno D.. (2020). Production of IL-17A at Innate Immune Phase Leads to Decreased Th1 Immune Response and Attenuated Host Defense against Infection with Cryptococcus deneoformans. J. Immunol. 205, 686–698. doi: 10.4049/jimmunol.1901238, PMID: 32561568

[B132] Schmidt S. Tramsen L. Lehrnbecher T. (2017). Natural killer cells in antifungal immunity. Front. Immunol. 8. doi: 10.3389/fimmu.2017.01623, PMID: 29213274 PMC5702641

[B133] Schulze B. Piehler D. Eschke M. von Buttlar H. Köhler G. Sparwasser T.. (2014). CD4(+) FoxP3(+) regulatory T cells suppress fatal T helper 2 cell immunity during pulmonary fungal infection. Eur. J. Immunol. 44, 3596–3604. doi: 10.1002/eji.201444963, PMID: 25187063

[B134] Scriven J. E. Graham L. M. Schutz C. Scriba T. J. Wilkinson K. A. Wilkinson R. J.. (2016). A glucuronoxylomannan-associated immune signature, characterized by monocyte deactivation and an increased interleukin 10 level, is a predictor of death in cryptococcal meningitis. J. Infect. Dis. 213, 1725–1734. doi: 10.1093/infdis/jiw007, PMID: 26768248 PMC4857465

[B135] Speakman E. A. Dambuza I. M. Salazar F. Brown G. D. (2020). T cell antifungal immunity and the role of C-type lectin receptors. Trends Immunol. 41, 61–76. doi: 10.1016/j.it.2019.11.007, PMID: 31813764 PMC7427322

[B136] Stempinski P. R. Ramos Irizarry P. McConnell S. A. Liporagi Lopes L. C. Rodrigues Dos Santos Júnior S. Wear M. P.. (2025). A Cryptococcus neoformans polysaccharide conjugate vaccine made with filtered polysaccharide elicits protective immunity in mice. Fungal Biol. 129, 101544. doi: 10.1016/j.funbio.2025.101544, PMID: 40023532 PMC13316834

[B137] Subramaniam K. Metzger B. Hanau L. H. Guh A. Rucker L. Badri S.. (2009). IgM(+) memory B cell expression predicts HIV-associated cryptococcosis status. J. Infect. Dis. 200, 244–251. doi: 10.1086/599318, PMID: 19527168 PMC2805277

[B138] Sun D. Zhang M. Liu G. Wu H. Li C. Zhou H.. (2016). Intravascular clearance of disseminating Cryptococcus neoformans in the brain can be improved by enhancing neutrophil recruitment in mice. Eur. J. Immunol. 46, 1704–1714. doi: 10.1002/eji.201546239, PMID: 27109176 PMC5165700

[B139] Sun D. Zhang M. Sun P. Liu G. Strickland A. B. Chen Y.. (2020). VCAM1/VLA4 interaction mediates Ly6Clow monocyte recruitment to the brain in a TNFR signaling dependent manner during fungal infection. PloS Pathog. 16, e1008361. doi: 10.1371/journal.ppat.1008361, PMID: 32101593 PMC7062284

[B140] Szymczak W. A. Davis M. J. Lundy S. K. Dufaud C. Olszewski M. Pirofski L. A. (2013). X-linked immunodeficient mice exhibit enhanced susceptibility to Cryptococcus neoformans Infection. mBio 4 (4), e00265-13. doi: 10.1128/mBio.00265-13, PMID: 23820392 PMC3705448

[B141] Trevijano-Contador N. de Oliveira H. C. Malacatus-Bravo C. Sarai V. Cuesta I. Rodrigues M. L.. (2025). Effects of human immunoglobulin A on Cryptococcus neoformans morphology and gene expression. Microbiol. Spectr. 13, e0200824. doi: 10.1128/spectrum.02008-24, PMID: 39982066 PMC11960444

[B142] Trevijano-Contador N. Pianalto K. M. Nichols C. B. Zaragoza O. Alspaugh J. A. Pirofski L. A. (2020). Human igM inhibits the formation of titan-like cells in cryptococcus neoformans. Infect. Immun. 88 (4), e00046-20. doi: 10.1128/iai.00046-20, PMID: 31988178 PMC7093138

[B143] Tucker S. C. Casadevall A. (2002). Replication of Cryptococcus neoformans in macrophages is accompanied by phagosomal permeabilization and accumulation of vesicles containing polysaccharide in the cytoplasm. Proc. Natl. Acad. Sci. U.S.A. 99, 3165–3170. doi: 10.1073/pnas.052702799, PMID: 11880650 PMC122490

[B144] Ueno K. Miyazaki Y. (2023). Detrimental impact of the IL-33/ST2 axis in an animal infection model with Cryptococcus neoformans. Allergol Int. 72, 530–536. doi: 10.1016/j.alit.2023.07.002, PMID: 37482531

[B145] Ueno K. Nagamori A. Honkyu N. O. Kwon-Chung K. J. Miyazaki Y. (2025). Lung-resident memory Th2 cells regulate pulmonary cryptococcosis by inducing type-II granuloma formation. Mucosal Immunol. 18, 631–642. doi: 10.1016/j.mucimm.2025.02.004, PMID: 39984054 PMC12167160

[B146] Ueno K. Yanagihara N. Otani Y. Shimizu K. Kinjo Y. Miyazaki Y. (2019). Neutrophil-mediated antifungal activity against highly virulent Cryptococcus gattii strain R265. Med. Mycol 57, 1046–1054. doi: 10.1093/mmy/myy153, PMID: 30668754

[B147] Uezu K. Kawakami K. Miyagi K. Kinjo Y. Kinjo T. Ishikawa H.. (2004). Accumulation of gammadelta T cells in the lungs and their regulatory roles in Th1 response and host defense against pulmonary infection with Cryptococcus neoformans. J. Immunol. 172, 7629–7634. doi: 10.4049/jimmunol.172.12.7629, PMID: 15187143

[B148] Velasco-de Andrés M. Català C. Casadó-Llombart S. Martínez-Florensa M. Simões I. García-Luna J.. (2020). The lymphocytic scavenger receptor CD5 shows therapeutic potential in mouse models of fungal infection. Antimicrob. Agents Chemother. 65 (1), e01103-20. doi: 10.1128/aac.01103-20, PMID: 33046489 PMC7927855

[B149] Velasco-de-Andrés M. Català C. Casadó-Llombart S. Simões I. Zaragoza O. Carreras E.. (2021). The lymphocyte scavenger receptor CD5 plays a nonredundant role in fungal infection. Cell Mol. Immunol. 18, 498–500. doi: 10.1038/s41423-020-0434-7, PMID: 32332900 PMC8027796

[B150] Vernel-Pauillac F. Laurent-Winter C. Fiette L. Janbon G. Aimanianda V. Dromer F. (2024). Cryptococcus neoformans infections: aspartyl protease potential to improve outcome in susceptible hosts. mBio 15, e0273324. doi: 10.1128/mbio.02733-24, PMID: 39440979 PMC11559057

[B151] Voelz K. Johnston S. A. Smith L. M. Hall R. A. Idnurm A. May R. C. (2014). 'Division of labour' in response to host oxidative burst drives a fatal Cryptococcus gattii outbreak. Nat. Commun. 5, 5194. doi: 10.1038/ncomms6194, PMID: 25323068 PMC4208095

[B152] Walsh N. M. Wuthrich M. Wang H. Klein B. Hull C. M. (2017). Characterization of C-type lectins reveals an unexpectedly limited interaction between Cryptococcus neoformans spores and Dectin-1. PloS One 12, e0173866. doi: 10.1371/journal.pone.0173866, PMID: 28282442 PMC5345868

[B153] Wang R. Oliveira L. V. N. Hester M. M. Carlson D. Christensen D. Specht C. A.. (2024). Protection against experimental cryptococcosis elicited by Cationic Adjuvant Formulation 01-adjuvanted subunit vaccines. PLoS Pathogens 20 (7), e1012220. doi: 10.1101/2024.04.24.591045, PMID: 38976694 PMC11257399

[B154] Wang Y. Pawar S. Dutta O. Wang K. Rivera A. Xue C. (2022). Macrophage mediated immunomodulation during cryptococcus pulmonary infection. Front. Cell Infect. Microbiol. 12. doi: 10.3389/fcimb.2022.859049, PMID: 35402316 PMC8987709

[B155] Wang S. Zhang Q. Hui H. Agrawal K. Karris M. A. Y. Rana T. M. (2020). An atlas of immune cell exhaustion in HIV-infected individuals revealed by single-cell transcriptomics. Emerg. Microbes Infect. 9, 2333–2347. doi: 10.1080/22221751.2020.1826361, PMID: 32954948 PMC7646563

[B156] Watanabe T. Nagai M. Ishibashi Y. Iwasaki M. Mizoguchi M. Nagata M.. (2025). Vacuolar sterol β-glucosidase EGCrP2/Sgl1 deficiency in Cryptococcus neoformans: Dysfunctional autophagy and Mincle-dependent immune activation as targets of novel antifungal strategies. PloS Pathog. 21, e1013089. doi: 10.1371/journal.ppat.1013089, PMID: 40273119 PMC12061408

[B157] Wiseman J. C. Ma L. L. Marr K. J. Jones G. J. Mody C. H. (2007). Perforin-dependent cryptococcal microbicidal activity in NK cells requires PI3K-dependent ERK1/2 signaling. J. Immunol. 178, 6456–6464. doi: 10.4049/jimmunol.178.10.6456, PMID: 17475875

[B158] Wozniak K. L. (2018). Interactions of cryptococcus with dendritic cells. J. Fungi (Basel) 4 (1), 36. doi: 10.3390/jof4010036, PMID: 29543719 PMC5872339

[B159] Wozniak K. L. Kolls J. K. Wormley F. L. Jr. (2012). Depletion of neutrophils in a protective model of pulmonary cryptococcosis results in increased IL-17A production by γδ T cells. BMC Immunol. 13, 65. doi: 10.1186/1471-2172-13-65, PMID: 23216912 PMC3538069

[B160] Xu J. Flaczyk A. Neal L. M. Fa Z. Eastman A. J. Malachowski A. N.. (2017). Scavenger receptor MARCO orchestrates early defenses and contributes to fungal containment during cryptococcal infection. J. Immunol. 198, 3548–3557. doi: 10.4049/jimmunol.1700057, PMID: 28298522 PMC5423401

[B161] Xu J. Ganguly A. Zhao J. Ivey M. Lopez R. Osterholzer J. J.. (2021). CCR2 signaling promotes brain infiltration of inflammatory monocytes and contributes to neuropathology during cryptococcal meningoencephalitis. mBio 12, e0107621. doi: 10.1128/mBio.01076-21, PMID: 34311579 PMC8406332

[B162] Xu L. Guo Y. Zhao Y. Xu Y. Peng X. Yang Z.. (2019). Chemokine and cytokine cascade caused by skewing of the th1-th2 balance is associated with high intracranial pressure in HIV-associated cryptococcal meningitis. Mediators Inflammation 2019, 2053958. doi: 10.1155/2019/2053958, PMID: 32082071 PMC7012228

[B163] Xu J. Hissong R. Bareis R. Creech A. Goughenour K. D. Freeman C. M.. (2024). Batf3-dependent orchestration of the robust Th1 responses and fungal control during cryptococcal infection, the role of cDC1. mBio 15, e0285323. doi: 10.1128/mbio.02853-23, PMID: 38349130 PMC10936214

[B164] Yang D. H. England M. R. Salvator H. Anjum S. Park Y. D. Marr K. A.. (2021). Cryptococcus gattii species complex as an opportunistic pathogen: underlying medical conditions associated with the infection. mBio 12, e0270821. doi: 10.1128/mBio.02708-21, PMID: 34700378 PMC8546560

[B165] Yang C. Gong Y. Liu S. Sun C. Wang T. Chen X.. (2024a). LincR-PPP2R5C deficiency enhancing the fungicidal activity of neutrophils in pulmonary cryptococcosis is linked to the upregulation of IL-4. mBio 15, e0213024. doi: 10.1128/mbio.02130-24, PMID: 39287443 PMC11481880

[B166] Yang C. Huang Y. Zhou Y. Zang X. Deng H. Liu Y.. (2022). Cryptococcus escapes host immunity: What do we know? Front. Cell Infect. Microbiol. 12. doi: 10.3389/fcimb.2022.1041036, PMID: 36310879 PMC9606624

[B167] Yang C. Shen W. Wang L. Zang X. Huang Y. Deng H.. (2024b). Cryptococcus gattii strains with a high phagocytosis phenotype by macrophages display high pathogenicity at the early stage of infection in *vivo* . Acta Biochim. Biophys. Sin. (Shanghai) 56, 291–303. doi: 10.3724/abbs.2023250, PMID: 37885429 PMC10984874

[B168] Zaragoza O. (2019). Basic principles of the virulence of Cryptococcus. Virulence 10, 490–501. doi: 10.1080/21505594.2019.1614383, PMID: 31119976 PMC6550552

[B169] Zhang T. Kawakami K. Qureshi M. H. Okamura H. Kurimoto M. Saito A. (1997). Interleukin-12 (IL-12) and IL-18 synergistically induce the fungicidal activity of murine peritoneal exudate cells against Cryptococcus neoformans through production of gamma interferon by natural killer cells. Infect. Immun. 65, 3594–3599. doi: 10.1128/iai.65.9.3594-3599.1997, PMID: 9284124 PMC175511

[B170] Zhang Y. Wang F. Bhan U. Huffnagle G. B. Toews G. B. Standiford T. J.. (2010). TLR9 signaling is required for generation of the adaptive immune protection in Cryptococcus neoformans-infected lungs. Am. J. Pathol. 177, 754–765. doi: 10.2353/ajpath.2010.091104, PMID: 20581055 PMC2913381

[B171] Zhang L. Zhang K. Li H. Coelho C. de Souza Gonçalves D. Fu M. S.. (2021). Cryptococcus neoformans-Infected Macrophages Release Proinflammatory Extracellular Vesicles: Insight into Their Components by Multi-omics. mBio 12 (2), e00279-21. doi: 10.1128/mBio.00279-21, PMID: 33785616 PMC8092229

[B172] Zhao X. Q. Zhu L. L. Chang Q. Jiang C. You Y. Luo T.. (2014). C-type lectin receptor dectin-3 mediates trehalose 6,6'-dimycolate (TDM)-induced Mincle expression through CARD9/Bcl10/MALT1-dependent nuclear factor (NF)-κB activation. J. Biol. Chem. 289, 30052–30062. doi: 10.1074/jbc.M114.588574, PMID: 25202022 PMC4208012

[B173] Zhou J. Lu X. He R. Du Y. Zeng B. Feng L.. (2025). Antifungal immunity: advances in PRR recognition, adaptive responses, and immune-based therapies. Sci. China Life Sci. 68 (8), 2206–2224. doi: 10.1007/s11427-024-2835-y, PMID: 40055278

[B174] Zhu F. Zhou Z. Ma S. Xu Y. Tan C. Yang H.. (2024). Design of a cryptococcus neoformans vaccine by subtractive proteomics combined with immunoinformatics. Int. Immunopharmacol 135, 112242. doi: 10.1016/j.intimp.2024.112242, PMID: 38772296

[B175] Ziegler-Heitbrock L. Ancuta P. Crowe S. Dalod M. Grau V. Hart D. N.. (2010). Nomenclature of monocytes and dendritic cells in blood. Blood 116, e74–e80. doi: 10.1182/blood-2010-02-258558, PMID: 20628149

